# Computationally-guided exchange of substrate selectivity motifs in a modular polyketide synthase acyltransferase

**DOI:** 10.1038/s41467-021-22497-2

**Published:** 2021-04-13

**Authors:** Edward Kalkreuter, Kyle S. Bingham, Aaron M. Keeler, Andrew N. Lowell, Jennifer J. Schmidt, David H. Sherman, Gavin J. Williams

**Affiliations:** 1 Department of Chemistry, NC State University, Raleigh, NC USA; 2 Comparative Medicine Institute, NC State University, Raleigh, NC USA; 3 Life Sciences Institute, Department of Medicinal Chemistry, University of Michigan, Ann Arbor, MI USA; 4 Department of Chemistry, Department of Microbiology & Immunology, University of Michigan, Ann Arbor, MI USA; 5 Present Address: Department of Chemistry, The Scripps Research Institute, Jupiter, FL USA; 6 Present Address: UNC Chapel Hill School of Medicine, Chapel Hill, NC USA; 7 Present Address: Department of Chemistry, Virginia Tech, Blacksburg, VA USA

**Keywords:** Enzymes, Natural products, Natural product synthesis, Molecular dynamics

## Abstract

Polyketides, one of the largest classes of natural products, are often clinically relevant. The ability to engineer polyketide biosynthesis to produce analogs is critically important. Acyltransferases (ATs) of modular polyketide synthases (PKSs) catalyze the installation of malonyl-CoA extenders into polyketide scaffolds. ATs have been targeted extensively to site-selectively introduce various extenders into polyketides. Yet, a complete inventory of AT residues responsible for substrate selection has not been established, limiting the scope of AT engineering. Here, molecular dynamics simulations are used to prioritize ~50 mutations within the active site of EryAT6 from erythromycin biosynthesis, leading to identification of two previously unexplored structural motifs. Exchanging both motifs with those from ATs with alternative extender specificities provides chimeric PKS modules with expanded and inverted substrate specificity. Our enhanced understanding of AT substrate selectivity and application of this motif-swapping strategy are expected to advance our ability to engineer PKSs towards designer polyketides.

## Abstract (web summary)

Engineering efforts have focused on acyltransferase (AT) domains of modular polyketide synthases (PKSs) to site-selectively modify the resulting polyketides, but critical AT residues involved in substrate selection have not been fully elucidated. Here, the authors use molecular dynamics to pinpoint mutations that impact AT domain selectivity and exchange structural motifs to obtain chimeric PKS modules with expanded substrate specificity.

## Introduction

Natural products are well-known for their clinical efficacy and their intricate biosynthetic pathways are frequently studied^1,2^. However, due to their structural complexity, chemical modification to yield new-to-nature natural product analogs is rarely simple or efficient^3^. Instead, engineering the biosynthesis of these valuable compounds and their analogs is often preferred^3^. Polyketides, one of the most prevalent classes of natural products, are responsible for tens of billions of dollars annually for antimicrobials^4^. Yet, designer polyketide analogs have not been easily accessible for this market, in large part due to difficulties in engineering the many different chemistries required for their biosyntheses.

**Fig. 1:**
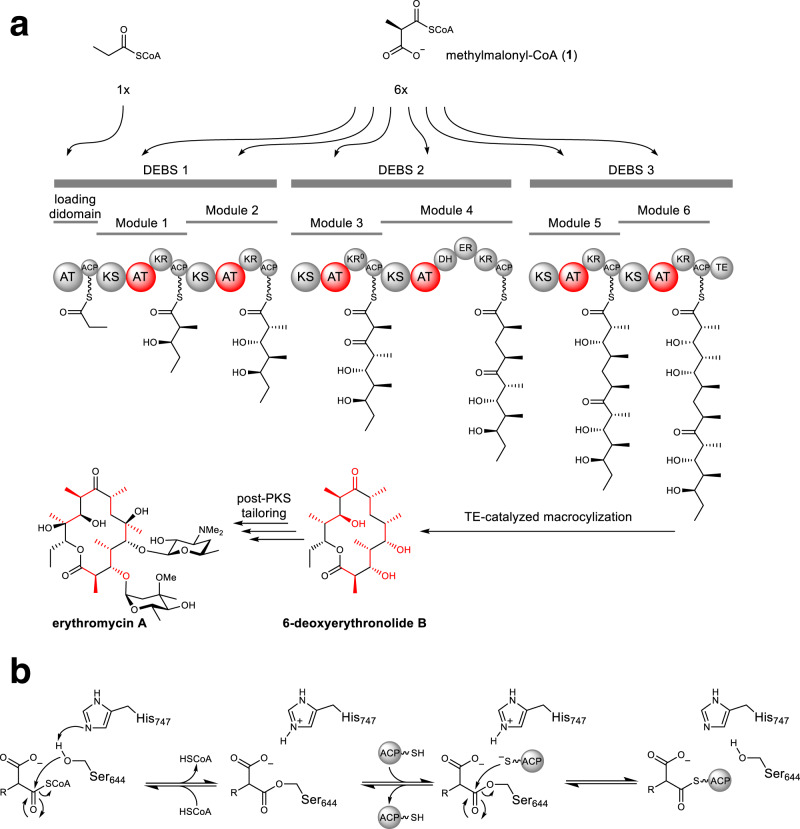
Organization of type I PKSs and mechanism of extender unit installation. **a** Domain and module organization of the prototypical type I PKS, DEBS. Each acyltransferase (AT) that selects an extender unit is shown in red, as is the contribution of each extender unit to the final polyketide structure. ACP acyl carrier protein, KS ketosynthase, KR ketoreductase, DH dehydratase, ER enoylreductase, TE thioesterase. **b** Abbreviated mechanism accounting for the installation of an extender unit onto the cognate ACP by EryAT6.

For example, Type I polyketide synthases (PKSs) are giant multi-modular enzymes responsible for the biosynthesis of the scaffolds of many clinically relevant polyketides through the controlled stepwise assembly of α-substituted malonyl-CoA extender units^4^. For canonical PKSs such as the prototypical 6-deoxyerythronolide B synthase (DEBS) from erythromycin biosynthesis, acyltransferase (AT) domains within each module are responsible for the selection of each extender unit and subsequent loading onto the cognate acyl carrier protein (ACP) (Fig. 1). The ketosynthase (KS) domain from each module can then catalyze the decarboxylative condensation between the ACP-linked extender unit and the extended chain from the prior module^5–7^. The most common extender units are malonyl-CoA (mCoA) and methylmalonyl-CoA (mmCoA, **1**), while ethylmalonyl-CoA (**2a**) and a few others are less frequently incorporated^8^. A large portion of the polyketide structure derives from extender units, therefore contributing significantly to their potent biological activity and pharmacological properties^3^. Accordingly, there is much interest in manipulating the extender unit selectivity of PKS modules to achieve the site-selective modification of polyketides. To this end, AT-swapping, motif-based chimeragenesis, and point mutagenesis have been explored to manipulate extender unit selection by PKSs^3,9–12^. However, AT-swapped PKSs typically produce the desired polyketide in poor yields or are completely inactive, likely due to disruption of critical protein interactions and substrate channeling^3^. Motif-swapping aims to exchange short sequences of residues that confer substrate selectivity. However, only a few conserved motifs have been identified and characterized, most notably the YASH motif (Supplementary Fig. 1)^13^. Usually, replacement of the YASH motif produces only a modest change in extender unit specificity, indicating that motif-swapped hybrids are disrupted in some way and/or that the current motifs do not capture all of the specificity-conferring residues^12,14^. For example, the active sites of AT2 and AT3 from the epothilone PKS differ by only nine residues, but the corresponding substrate selectivity is mCoA in AT2 and is relaxed towards mCoA/mmCoA in AT3. The YASH motif alone is not responsible for this difference, even though it accounts for two of the nine amino differences between the two active sites^10,15^. In contrast to exchanging residues between ATs, point mutations in or near the YASH motif of ATs of the DEBS (EryAT6) or pikromycin (PikAT5/PikAT6) pathways enable incorporation of non-natural extender units and significant changes to substrate selectivity without the deleterious effects of domain/module swapping^16–20^. Yet, not all of these first-generation mutations are transferable between ATs in different PKSs^20^. To be maximally efficient and broadly applicable, motif-swapping and point mutagenesis would benefit from a comprehensive inventory of AT residues responsible for substrate selection. Moreover, although the chemical diversity of naturally occurring extender units is somewhat limited, ATs that accept rare ones provide a potential template for engineering other PKSs to accept non-native or by extension, non-natural extender units.

Here, a methodology guided by molecular dynamics (MD) simulations was employed to identify and mutate specificity-conferring residues within the AT domain, using EryAT6 as a model system. Collectively, these data informed additional predictions and enabled further exploration of a recently identified ketoreductase (KR) domain bottleneck^20^. Consequently, nine residues previously unassociated with AT substrate selectivity were identified. Insights from MD studies and analysis of EryAT6 mutations culminated in the prioritization of two previously unexplored structural motifs within the AT active site. Substitution of both motifs in EryAT6 with those from unrelated ATs with unusual extender unit specificities provided chimeric PKS modules with expanded and inverted substrate scope. These improvements in our understanding of AT substrate selectivity is expected to advance our ability to engineer PKSs towards an eventual goal of designer polyketides.

## Results

### Molecular dynamics simulations of wild-type and first-generation engineered EryAT6 variants

**Fig. 2:**
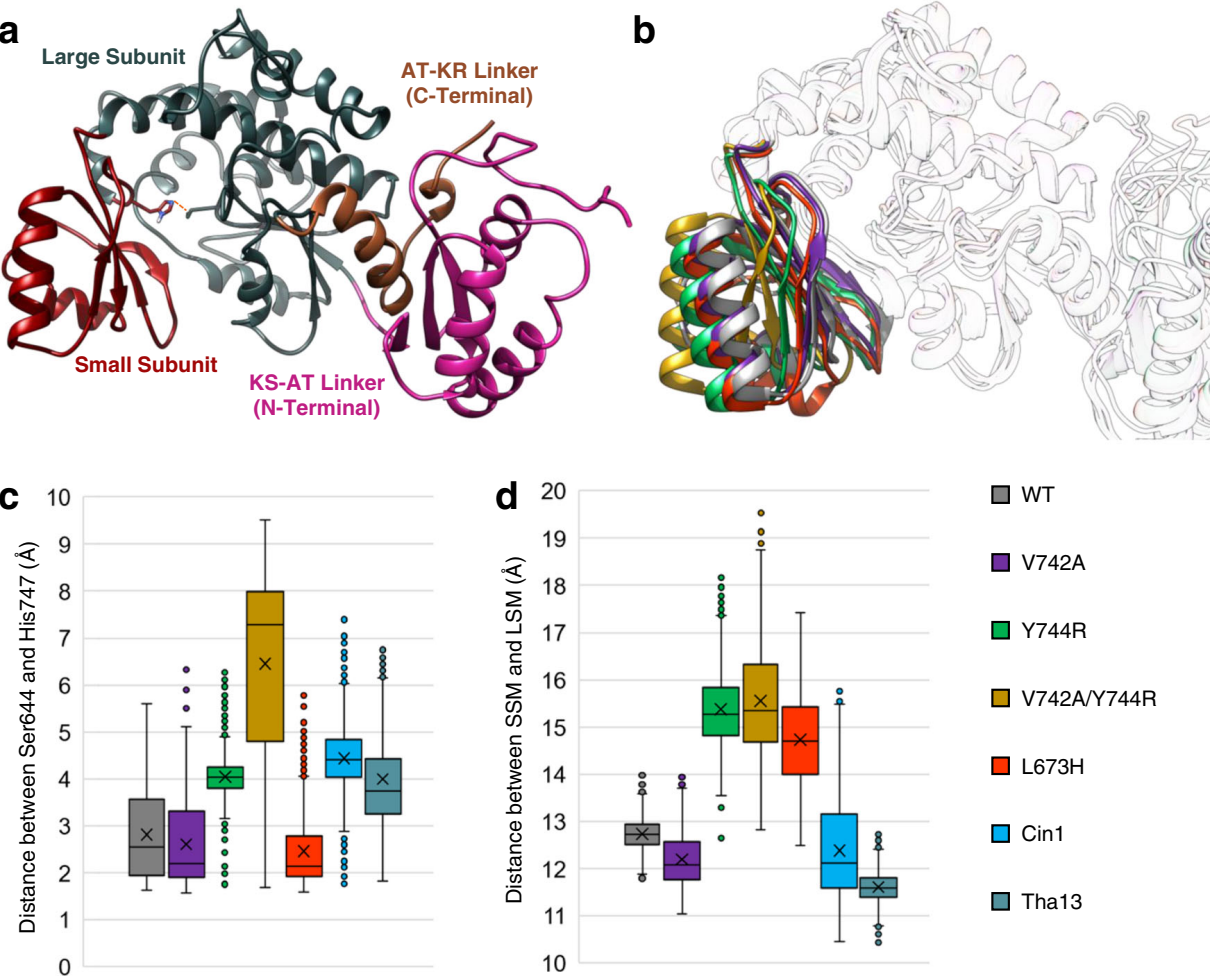
Molecular dynamic simulations of wild-type and variant EryAT6. **a** Final homology model of EryAT6 containing both linkers. The catalytic dyad is shown as sticks with the distance between indicated with a dashed orange line. **b** Overlay of time-averaged structures from the EryAT6 wild-type and mutant simulations highlighting the distortion of the small subunit. **c** A box plot shows how the distance between the catalytic dyad varied over simulation time for each EryAT6 variant. **d** A box plot shows how the distance between the center of mass for the SSM and LSM varied over simulation time for each EryAT6 variant. The *X* indicates the mean. The boxes represent the range from the first to third quartiles with the center line representing the median. The whiskers represent the minima and maxima or, in the presence of outliers indicated by points, 1.5 times the interquartile range. These distances were measured every 0.1 ns over the final 80 ns (*n* = 800) of two independent simulations. WT = wild-type. Source data are provided as a Source Data file.

**Fig. 3:**
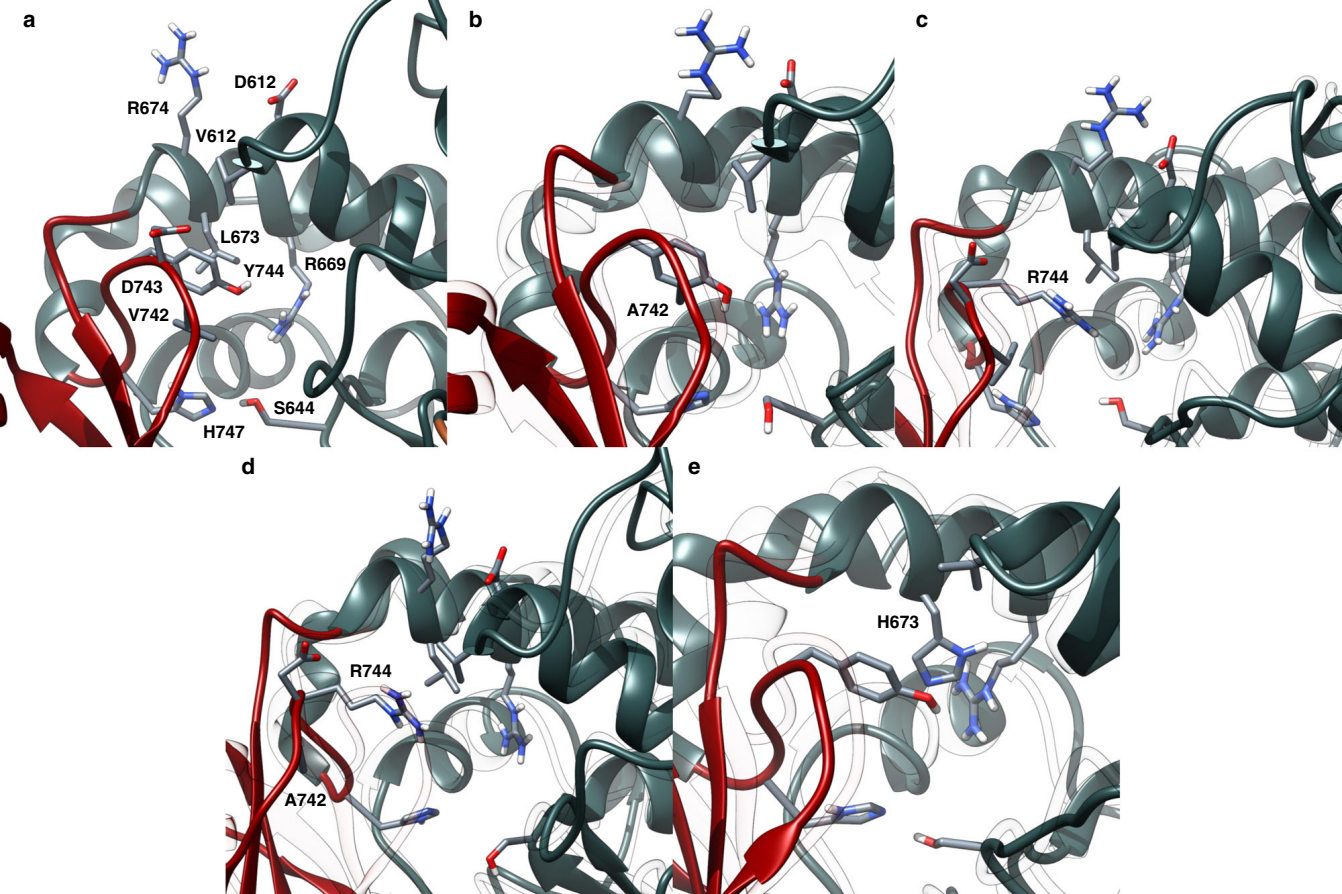
Snapshots from EryAT6 simulations showing key residues with the active site. **a** Wild-type EryAT6 shows the catalytic dyad His747 and Ser644, hydrophobic interactions between Tyr744, Val742, Leu673, and Val612, and transient charged interactions between Arg674, Asp612, and Asp743. Arg669 positions the carboxylate of the substrate for catalysis. The wild-type backbone is shown as a transparent overlay with all mutants. **b** The V742A mutation results in a weaker interaction between Ala742 and Tyr744 than in wild-type, as well as less bulkiness within the active site, but the overall structure remains similar to wild-type. **c** The Y744R mutant introduces transient charged interactions between Arg744, Asp743, and Asp613 while disrupting the wild-type hydrophobic interactions. **d** The double mutant V742A/Y744R combines the features of the two individual mutants, resulting in a much wider active site. **e** The L673H mutant results in π-stacking between Tyr744 and His673, creating a narrower active site and disrupting the interaction between Tyr744 and Val612. Time-averaged structures are shown Supplementary Fig. S5. Y744R mutation impacts the salt bridge, resulting in a larger inter-subunit distance. The wild-type model is overlaid as transparent ribbons. **c** Wild-type EryAT6 showing few interactions between Tyr744 and the large subunit. (**d**) Introduction of the L673H mutation results in a narrower active site via π-stacking between Tyr744 and His673. The wild-type model is overlaid as transparent ribbons.

To obtain an enhanced understanding of how EryAT6 selects its extender unit for transfer to the ACP, MD simulations of the wild-type EryAT6 were carried out and compared to those containing previously identified substrate selectivity-shifting mutations, V742A, Y744R, V742A/Y744R, and L673H^16,18,21^. The first three of these mutations shifts selectivity of Ery6TE towards propargylmalonyl-CoA while the fourth mutation shifts selectivity towards mmCoA (wild-type Ery6TE has some native promiscuity)^16,18,22^. A small panel of EryAT6 homology models was first constructed that included different combinations of the N- and C-terminal interdomain linkers with the core AT structure (Supplementary Fig. 2). These models were subjected to 50 ns MD simulations, and the simulations were analyzed for stability of secondary structure (Supplementary Fig. 3), RMSD (Supplementary Fig. 4a–c), and maintenance of a reasonable distance between the catalytic dyad of Ser644 and His747, which are located on opposite sides of the hinge-like active site, within the large and small subunits of the AT, respectively (Fig. 2a). This distance was used to approximate the width of the active site and likely determines if the enzyme is in a catalytically competent state. The final model, with a 109-residue N-terminal KS-AT linker and a 43-residue C-terminal AT-KR linker flanking the 278-residue core AT (Fig. 2a), is in good agreement with an earlier study on AT boundaries (Supplementary Table 1)^14^, and it retained the expected secondary structure better than in the models lacking one or both linkers. This wild-type EryAT6 model was solvated and simulated over 100 ns (Supplementary Fig. 4c). This structure was also used as the basis for all subsequent point mutation simulations, including the introduction of the individual mutations V742A, Y744R, and L673H, and the double mutation V742A/Y744R^16,18^. For each simulation trajectory, RMSD (Supplementary Fig. 4), structural rearrangements (time-averaged structures and individual snapshots, Figs. 2b, 3, and Supplementary Fig. 5), residue-level movement and flexibility (root-mean-square fluctuation, RMSF, Supplementary Fig. 6), and the distance between residues in the two subunits (Fig. 2c, d) were inspected.

The average structure of the V742A mutant is very similar to that of wild-type in the simulation (Fig. 2b and Supplementary Fig. 5a). In an attempt to quantify the effect of the mutation, the distance between the two catalytic residues, positioned across the active site and in opposite subunits, was used as a proxy for active site width. Perhaps surprisingly, while the Ser644-His747 distance correlated well with acceptance of larger substrates (Fig. 2c), the distance between Ser644 and Ser746, the third residue of the YASH motif, showed little predictive value for AT selectivity (Supplementary Fig. 7a). However, by this dyad measurement, the V742A mutant was nearly indistinguishable from wild-type (average of 2.815 Å for wild-type and 2.609 Å for V742A). Instead, the enhanced selectivity towards larger substrates is likely due to a combination of reduced sterics (Fig. 3a, b) and increased movement of a major portion of the small subunit resulting from the greater freedom of movement of the active site loop containing Ala742 (Supplementary Fig. 6a). It is possible that this greater freedom was due in part to a reduced hydrophobic interaction between Ala742 and the nearby Tyr744.

In contrast to the relatively minor changes seen in the V742A mutant, the Y744R mutant simulation presents much more significant perturbations to the AT that correlate well with its inverted substrate selectivity^16^. Major changes might be expected after the introduction of an Arg into the active site, but it is not an obvious mutation to introduce to increase selectivity towards a larger substrate. In this case, the simulation highlights the hinge-like nature of the AT, with the large and small subunits moving back and forth to create a dynamic active site (Fig. 2b, c). In the Y744R mutant, the enzyme can still access wider points than the wild-type and V742A mutant can (>5 Å dyad distance), but it cannot access dyad distances under 3 Å (average of 4.042 Å). In addition to creating more space around the catalytic dyad, the distance between the two subunits (by center of mass) shifts with this mutant by about 2 Å (Supplementary Fig. 7b). Selected snapshots throughout the simulation hint at a mechanism for arresting the hinge-like action of the AT: (i) Arg744 can disrupt the salt bridge between Arg674 of the large subunit and Asp743 of the small subunit; and (ii) Arg744 extends into the active site, directly towards and adjacent to Val612 and Leu673, respectively, of the large subunit, preventing the subunits from moving closer together, in place of the hydrophobic interactions seen between Tyr744 and Val612 in the wild-type (Fig. 3a, c).

In our previous study, the combination of the V742A and Y744R mutations resulted in a poorly active, but highly propargyl-selective, AT^16^. In order to move forward with engineering ATs with orthogonal selectivities, any synergy between individual mutations needs to be understood. Gratifyingly, an initial examination of the simulation of the double mutant could rationalize both its selectivity and its poor activity. The time-averaged structure of the double mutant stands out from all other structures by several angstroms (Fig. 2b), and the dyad distance (average of 6.455 Å; Fig. 2c) and distance between subunits (Supplementary Fig. 7b) shows how rarely the AT could access an active site capable of accepting mmCoA. Likewise, with the catalytic residues so distant from each other most of the time, the activity would necessarily be much lower than either of the individual mutants or wild-type. Snapshots from the trajectory showed a similar orientation of Arg744 as in the Y744R single mutant, but with the added flexibility attributed to the V742A mutation, the small subunit could swing out further to provide more space for the positively charged Arg744 (Fig. 3d).

In contrast, the mmCoA-specific L673H mutant replaces a hydrophobic leucine with a π-π stacking interaction between Tyr744 of the YASH motif and His673 of the large subunit (Fig. 3a, e). As expected, the dyad distance shrinks for this mutant slightly relative to wild-type (average 2.459 Å; Fig. 2c), but surprisingly, the distance between the two subunits increased by nearly as much as in the Y744R simulation relative to wild-type (Supplementary Fig. 7b; + 2.343 Å for Y744R; + 1.566 Å for L673H). Upon examination of selected snapshots (Fig. 3), this apparent incongruity could be explained due to a shared aspect of the L673H and Y744R mutations: disruption of the hydrophobic interactions between Tyr744, Val742, and Val612. In the wild-type AT, Tyr744 has stabilizing interactions with both residues, but in the L673H mutant, the π-stacking between Tyr744 and His673 draws the back of the enzyme closer together (resulting in a shorter dyad distance) while eliminating other interactions between the two subunits (resulting in a wider distance between the subunits).

### Probing the extender unit selectivity of wild-type Ery6TE via a multi-substrate competition assay

**Fig. 4:**
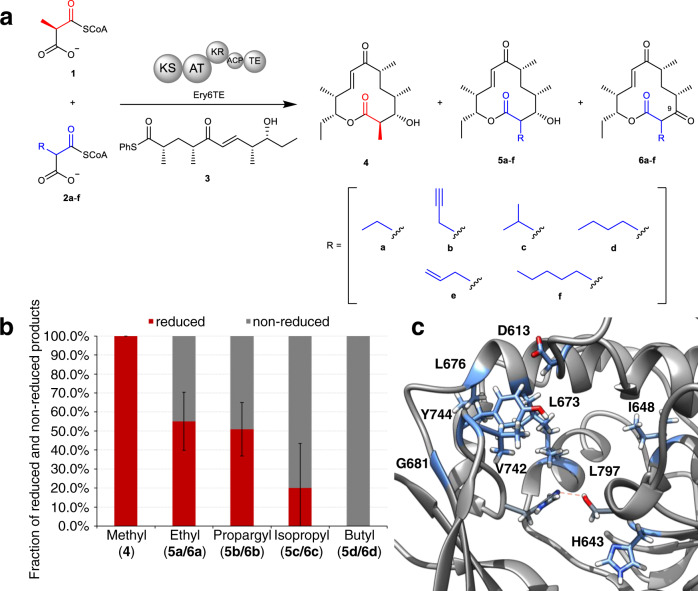
Ery6TE multi-substrate competition assay. **a** The Ery6TE module is incubated with the synthetic pentaketide chain mimic **3** and an equimolar mixture of the native extender **1** and mixtures of **2a**–**f** in vitro. Products **4** and **5a**–**f** are produced upon AT-catalyzed extender unit installation, KS-extension, KR-reduction, and cyclization by the TE. In contrast, **6a**–**f** are produced when the KR does not process the C-9 keto. NADPH was produced in situ with an NADPH regeneration system (not shown). **b** The fraction of each reduced (**4**, **5a**–**d**) and non-reduced (**6a**–**d**) product (total = 100) catalyzed by wild-type Ery6TE as determined by HR LC-MS analysis. Error bars are the standard deviation (*n* = 3 biological replicates) of the average fraction of the non-reduced (**6a**–**6d**) product. **c** The active site of wild-type EryAT6 showing key residues (in blue) that were targeted for individual mutations and successfully affected substrate selectivity.

Next, the in vitro extender unit selectivity of wild-type Ery6TE was determined using a pool of competing equimolar extender units (300 μM each of **1**, **2a**–**d**, Fig. 4a and Supplementary Fig. 8) and a previously described synthetic thiophenol-pentaketide substrate (**3**) based on the pikromycin PKS pathway^16,20,23^. An NADPH recycling system was included to support KR activity and production of the 10-deoxymethynolide (10-dML) series **4** and **5a**–**d** upon extender unit installation and cyclization. The corresponding non-reduced series **6a**–**d** are possible if the KR does not process the nascent β-carbonyl, as reported previously^20^. Subsequently, the percentage distribution of the products derived from each extender unit was determined by high-resolution liquid chromatography with tandem mass spectrometry (LC-MS) analysis. As anticipated, the major product in the wild-type Ery6TE competition assay was derived from the native extender **1** (**4**, 58%), followed by those derived from the propargyl-**2b** (27%), isopropyl-**2c** (11%), ethyl-**2a** (3%), and butyl-**2d** (1%) extenders (Table 1 and Supplementary Tables 2, and 3). Although wild-type Ery6TE displays significant in vitro extender unit promiscuity, some extenders are poorly utilized (e.g., **2b**, **2d**). Nevertheless, this baseline promiscuity serves as a good starting point to manipulate the extender unit specificity by protein engineering.

**Table 1:** Peak area distributions catalyzed by wild-type and variant Ery6TE in the presence of competing equimolar 300 μM extender units.

Ery6TE variant^a^	4^b^	5a + 6a^b^	5b + 6b^b^	5c + 6c^b^	5d + 6d^b^	Relative activity^c^
WT	57.7 ± 2.2%	2.9 ± 0.2%	27.1 ± 0.9%	11.4 ± 0.4%	1.0 ± 0.0%	100.0
D613E	56.4 ± 4.6%	2.5 ± 0.4%	25.9 ± 3.1%	13.4 ± 1.1%	1.9 ± 0.0%	101.9 ± 13.3
H643F	95.0 ± 2.9%	1.1 ± 0.0%	1.9 ± 0.2%	1.7 ± 0.2%	0.3 ± 0.0%	153.5 ± 4.3
H643S	52.9 ± 2.7%	4.5 ± 0.1%	25.5 ± 2.3%	13.1 ± 1.5%	4.1 ± 0.1%	57.6 ± 7.0
I648G	96.7 ± 2.1%	0.4 ± 0.1%	0.8 ± 0.1%	1.7 ± 0.2%	0.4 ± 0.0%	180.8 ± 12.8
L673G	65.7 ± 2.2%	2.5 ± 0.4%	22.9 ± 1.6%	6.5 ± 0.9%	2.4 ± 0.2%	152.6 ± 17.6
L676V	73.4 ± 1.6%	3.1 ± 0.1%	7.8 ± 0.9%	11.7 ± 1.0%	4.1 ± 0.5%	135.1 ± 23.9
G681A	59.5 ± 2.0%	2.7 ± 0.4%	18.6 ± 0.8%	17.8 ± 1.2%	1.5 ± 0.0%	124.6 ± 22.6
V742A	56.0 ± 2.4%	4.3 ± 0.2%	35.6 ± 2.4%	2.4 ± 0.2%	1.7 ± 0.2%	76.8 ± 1.8
Y744R	17.7 ± 1.2%	2.5 ± 0.1%	57.0 ± 3.2%	13.0 ± 1.9%	9.7 ± 0.2%	46.2± 4.1
V751A	54.3 ± 2.8%	2.7 ± 0.3%	29.7 ± 2.3%	10.7 ± 0.2%	2.5 ± 0.2%	76.5 ± 3.7
L797E	98.4 ± 1.3%	0.5 ± 0.1%	0.5 ± 0.0%	0.3 ± 0.0%	0.3 ± 0.0%	259.6 ± 47.0
Ery6(Cin1)TE	16.3 ± 0.2%	0.6 ± 0.1%	55.8 ± 5.9%	6.9 ± 0.5%	20.3 ± 2.7%	35.9 ± 5.7
Ery6(Tha13)TE	11.2 ± 0.2%	0.9 ± 0.0%	18.3 ± 0.8%	23.2 ± 3.0%	46.5 ± 2.9%	30.1 ± 1.4

^a^Module lysates were incubated with 300 μM each of **1**, **2a**, **2b**, **2c**, and **2d** simultaneously. Peak distributions of mutants that could not be distinguished from wild-type Ery6TE are omitted for clarity.

^b^Percent of each indicated peak area out of total product peak area (**4**, **5a**, **6a**, **5b**, **6b**, **5c**, **6c**, **5d**, and **6d**). Error bars are the standard deviation (*n* = 3 biological replicates) of the mean. See Fig. 3a for structures of products.

^c^Total activity of each system relative to wild-type Ery6TE is based on total peak areas of products and normalized to amount of enzyme (**4**, **5a**, **6a**, **5b**, **6b**, **5c**, **6c**, **5d**, and **6d**).

Notably, while wild-type Ery6TE appears to discriminate against the largest extender (butyl) and the extenders most similar to the native extender (Table 1 and Supplementary Tables 2 and 3), the fraction of the non-reduced product correlated to the size of the extender unit C-2 side-chain (Fig. 4b). For example, 100% of the product is reduced when the natural substrate **1** is utilized, while 100% of the product is non-reduced when derived from the butyl substrate **2d** (Fig. 4b), in a similar fashion to KR5 in an engineered pikromycin module^20^. While the KR likely plays some role in determining whether a given extender unit is converted into a final cyclized product by Ery6TE, clearly the specificity restrictions of the AT needs to be overcome first in order to provide the KR the opportunity to carry out both native and non-native C-9 reductions.

### Extensive mutagenesis of the Ery6AT active site

To test the hypothesis from the MD simulations that a large portion of residues in the AT active site contributes to extender unit selectivity, especially through modulation of the inter-subunit interactions, mutations at residues outside the YASH and other established motifs were designed by inspection of the EryAT6 model and simulations (Fig. 4c and Supplementary Table 4). Briefly, mutations were designed to influence extender unit selectivity based on predicted impact on subunit interactions and flexibility, direct substrate interactions, and active site size. In total, 32 mutants were constructed, spanning 26 residues. Included for comparison were three previously reported mutations: V742A, Y744G, and Y744R.^61,143^ Val742 and Tyr744 are located near and in the conserved YASH motif, respectively (Fig. 4c). Each mutant was probed using the multi-substrate competition assay as described for the wild-type enzyme (Table 1 and Fig. 4c).

For the first-generation mutants, the largest change in product distribution for V742A was the ethyl- (**5a** + **6a**) and butyl-macrolactones (**5d** + **6d**), with a 1.5- and 2.0-fold improvement compared to the wild-type, respectively. Nevertheless, similar to wild-type Ery6TE, the methyl-derived product (**4**) was the major product for V742A. Introduction of Y744R led to a 2- and 10-fold improved production of the propargyl- (**5b** + **6b**) and butyl-macrolactones (**5d** + **6d**), respectively, with the propargyl-macrolactones (**5b** + **6b**) being the major products. The product distributions of 21 mutants could not be distinguished from that of the wild-type (Supplementary Table 4). However, 8 designed mutants (H643F, H643S, I648G, L673G, L676V, G681A, V751A, L797E) led to changes in product distributions, indicating that their substrate selectivity’s were altered (Table 1). Although the **1**-derived macrolactone **4** was the major product for each of these single mutants, some supported up to ~4-fold increase in the proportion of products derived from non-native extenders. For example, the butyl products (**5d** + **6d**) were increased by 2.4-, 4.1-, 2.5-, and 1.9-fold with L673G, L676V, V751A, and D613E, respectively, compared to wild-type. Interestingly, substitution at His643 (Fig. 4c), which is expected to position the thioester of the malonyl-CoA substrate for catalysis, with serine or phenylalanine providing opposite effects on the product distributions. H643S improved the fraction of the ethyl (**5a** + **6a**) and butyl (**5d** + **6d**) products 1.6- and 4.1-fold, respectively, compared to the wild-type, while 95% of the products of the H643F mutant were from the native substrate **1**, and this mutant was more stringent than the wild-type (Table 1).

Two other **1**-specific mutations were identified. I648G and L797E both resulted in >95% methyl product **4** (Table 1). I648G is located outside the active site within the large subunit (Fig. 4c) and is proposed to increase the flexibility of the conserved GHSxG loop and thus shorten the distance between the catalytic dyad. In contrast, the introduction of the negative charge directly behind the hydrophobic Leu673 via L797E likely restricts space in the rear of the active site.

To summarize, mutations at five previously untargeted residues (H643S, L673G, L676V, G681A, V751A) impact selectivity for larger extenders, and mutations at three residues (H643F, I648G, L797E) increase specificity for the native substrate **1**. While only one of these residues (His643) likely directly interacts with the substrate, the overall findings highlight that residues in both subunits of the AT play a role in dictating specificity, albeit making minor contributions individually. As seen with the MD simulations (Fig. 2 and Supplementary Fig. 5), single mutations can cause significant structural changes throughout the active site. Accordingly, because most of these mutations implicate molecular networks outside the established YASH motif, a combination of rationally designed substitutions at multiple contiguous sites could realize more extreme selectivity shifts.

### Introduction of active site motifs to invert extender unit selectivity of Ery6TE

**Fig. 5:**
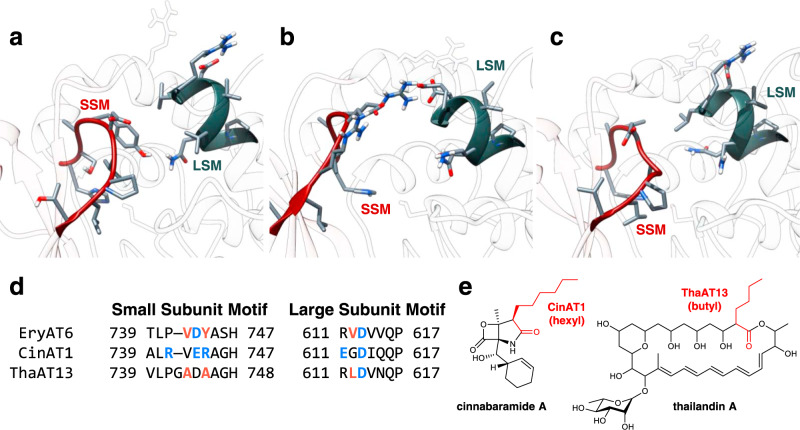
EryAT6 active site and chimeragenesis. Snapshots of the active sites of wild-type (**a**), Cin1 (**b**), and Tha13 (**c**) EryAT6 homology models from the 100 ns molecular dynamics simulations. The two structural motifs are represented by sticks and highlight the interactions between the large subunit (blue gray) and small subunit (red). Arg674 and the catalytic Ser644 are shown for their interactions with the two motifs. **d** Amino acid alignment of EryAT6, CinAT1, and ThaAT13, showing the identity of residues in the small subunit motif (SSM) and the large subunit motif (LSM). Indicated residues are expected to interact within the two motifs through hydrophobic (orange) and charge-mediated (blue) interactions. **e** Structures of the natural products biosynthesized by the cinnabaramide and thailandin PKS. Portions of the structures dictated by CinAT1 and ThaAT13 are highlighted in red.

The large conformational changes observed in the MD simulations, coupled with the established impact of mutations throughout the entire AT active site, suggest that targeting structural motifs could be more effective than single mutations by compensating for otherwise deleterious effects and maximizing synergy between subtle amino acid variations. Most notably, a loop in the small subunit spanning Thr739 to His747 (including the YASH motif) in Ery6TE, defined here as the small subunit motif (SSM; Fig. 5a, d), is the region that contains the most direct interactions with the substrate. Additionally, in vitro reactions and MD simulations implicated mutations within this loop (especially V742A and Y744R), to impact substrate specificity (Fig. 2 and Table 1). Substrate selectivity-shifting mutations at the nearby Gly681, Val751, Leu673, and Leu676, suggest that these residues also likely interact with this loop.

While the entirety of the SSM is not conserved by substrate specificity (Supplementary Fig. 9), it spans the full length of the active site, from the active site opening through to the back of the pocket, and therefore if targeted for motif-swapping would be expected to retain structural integrity while being able to better accommodate the CoA-linked substrate not only once bound, but also during its approach into the pocket. In addition, a second motif dubbed the large subunit motif (LSM), makes up the portion of the helix directly above the catalytic Ser644 in the large subunit, spanning from Arg611 to Pro617 in EryAT6, and contains the well-conserved mmCoA-specific VDVVQP residues (Fig. 5a, d and Supplementary Fig. 9b)^19,24^. Residues within this motif have been previously implicated in substrate selectivity, with the Q616H mutation increasing promiscuity in EryAT6 and a Phe residue shown to π-stack with a phenylmalonyl-CoA substrate in the splenocin PKS^19,24^. The LSM is not expected to have any obvious interactions with the C2-side-chain of most substrates. However, in several of the wild-type and mutant MD trajectories, residues from the SSM and the LSM interact directly (especially Tyr744 with Val612) or indirectly through Arg674 (with Asp743 and Asp613) to maintain the hinge-like movement of the AT without too much movement, as in the V742A/Y744R double mutant (Fig. 3). In support of these predicted interactions, residue differences in one motif often correspond to complementary changes in the other motif in many ATs of varying substrate specificities (Supplementary Fig. 9a). Therefore, the motifs are implicated as potential paired exchangeable structural unit that could be targeted to manipulate extender unit selection.

To test this hypothesis, the SSM and the LSM in EryAT6 were replaced with the corresponding residues from two unrelated ATs with different extender unit specificities (Fig. 5b–e), the hexylmalonyl-CoA-selective AT1 from the cinnabaramide PKS in *Streptomyces cinnabarigriseus* (CinAT1) and the butylmalonyl-CoA-selective AT13 from the thailandin PKS in *Actinokineospora bangkokensis* (ThaAT13)^25,26^, producing two chimeras, Ery6(Cin1)TE and Ery6(Tha13)TE, respectively. Each chimera now differs from the wild-type Ery6TE at nine and seven residues, respectively.

Each parent AT has one or more distinctive features in the two motifs, which are predicted to play major roles in their respective substrate selectivities. CinAT1 represents one of the rare natural ATs containing the equivalent Y744R mutation within the YASH motif, but it also introduces a second arginine into the SSM (Fig. 5b, d). Despite suggestions that an arginine at position 744 prevents efficient use of extenders much larger than propargyl^19^, CinAT1 uses larger extenders natively^25^. The two flexible and positively charged arginines are positioned to be compensated by one or both of two nearby glutamates, one in each motif (Fig. 5b, d). These charge-mediated interactions may replace the largely hydrophobic interactions between the two motifs in EryAT6 (involving Tyr744, Val742, and Val612, Fig. 5a, d) and ThaAT13 (involving Ala745, Ala743, and Leu612, Fig. 5c, d). The additional methylene group of Glu743 in the CinAT1 SSM also provides the side chain length required to position both arginines mostly out of the active site channel. This arrangement contrasts with that of Arg744 in the Y744R single mutant in EryAT6, where it is instead held in place between the two subunits (Fig. 3c) and clashes with the hydrophobic Val612, Leu673, and Val742. ThaAT13 has a similar LSM to EryAT6, but in its SSM, five of the ten residues are either alanine or glycine, resulting in a significantly smaller and more flexible loop than that found in EryAT6. In addition to AAGH replacing the YASH motif, Val742 in EryAT6 has been replaced with a glycine and an alanine in ThaAT13, providing an extra residue and increased flexibility and space within the active site (Supplementary Fig. 9). Unlike in the unstable V742A/Y744R mutant, the Tha13 motifs cooperatively compensate for the smaller residue (Ala743) via the extra length of ThaAT13’s Leu612 relative to EryAT6’s Val612, maintaining the stabilizing hydrophobic interaction that is replaced by charge-mediated interactions in CinAT1.

During 100 ns simulations of the two motif-swapped EryAT6 variants, the synergistic nature of these two motifs was further highlighted. Whereas in each single/double mutant the two subunits are forced apart and increased movement is seen across the AT, the two motif-swapped mutants show comparable inter-subunit distances and similar RMSF values to wild-type EryAT6 (Supplementary Figs. 6 and 7). Likewise, the distance between the SSM and LSM shows the retention of interactions in the motif-swapped ATs, unlike in the Y744R, L673H, and V742A/Y744R simulations (Fig. 2d). Critically, the motif swaps still provide the larger active sites needed for enhanced selectivity towards larger substrates, with both ATs having dyad distances longer than even the Y744R mutant (Fig. 2c).

**Fig. 6:**
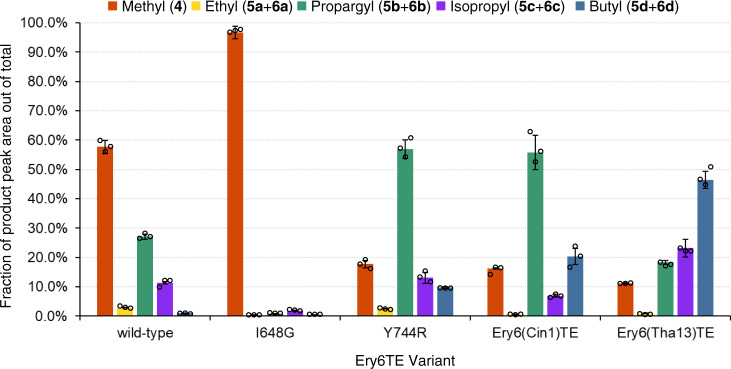
Product distributions catalyzed by motif-swapped Ery6TE variants with five competing substrates. Motif-swapped and selected individual mutant module lysates were incubated with 300 μM each of **1**, **2a**, **2b**, **2c**, and **2d**, simultaneously. Products were analyzed by HR-LC-MS, and the product distributions are shown. Error bars are the standard deviation (*n* = 3 biological replicates) of the mean. Individual replicates are shown as circles. Source data are provided as a Source Data file.

Based on this logic, the extender unit selectivity of the two motif-swapped Ery6TE chimeras was probed with the multi-substrate competition assay, revealing a large shift in preference towards larger extender units and mirroring the selectivity expected from the parent wild-type ATs (Fig. 6, Table 1, and Supplementary Table 3). For example, the propargyl (**5b** + **6b**) and butyl (**5d** + **6d**) products increased 2- and 20-fold, respectively, in the Ery6(Cin1)TE-catalyzed reaction, compared to wild-type Ery6TE. Concomitantly, the fraction of the methyl (**4**), ethyl (**5a** + **6a**), and isopropyl (**5c** + **6c**) products decreased 3.5-, 4.8-, and 1.7-fold, respectively, indicating a shift in substrate selectivity towards the propargyl- and butyl-extender units. Similarly, the Ery6(Tha13)TE chimera also provided a shift in selectivity towards the larger extender units, but the overall change is more pronounced. For instance, the fraction of the isopropyl (**5c** + **6c**) and butyl (**5d** + **6d**) products increased 2- and 47-fold, respectively, compared to wild-type Ery6TE, while that of the methyl (**4**), ethyl (**5a** + **6a**), and propargyl (**5b** + **6b**) products decreased 5-, 3.2-, and 1.5-fold, respectively, compared to wild-type Ery6TE. Subsequently, the product distribution of the Ery6(Tha13)TE chimera mirrors what is expected from the use of the motif from the butyl-**2d** utilizing ThaAT13. Interestingly, the optimal extender units for the Ery6(Cin1)TE chimera or CinAT1 are not yet known, but propargyl (**5b** + **6b**) production by Ery6(Cin1)TE is indistinguishable from that of Y744R (Fig. 6), while the fraction of the butyl product (**5d** + **6d**) supported by this chimera is more than double that of the single mutant Y744R. For comparison, the methyl-specific I648G mutant inspired by the MD simulations was included (Fig. 6) to emphasize the orthogonal AT substrate selectivities expected of the designed AT mutants. Crucially, in addition to the expected dramatic shifts in extender unit selectivities displayed by the motif-swapped chimeras, the relative activities compared of both chimeras are robust (>30%, Table 1) compared to the previously reported V742A/Y744R double mutant and many reported values for whole AT swaps^14,16^. These mutants, though retaining lower overall activity than some of the single mutants, are still comparable to the Y744R single mutant and show higher overall production with the largest substrates.

**Fig. 7:**
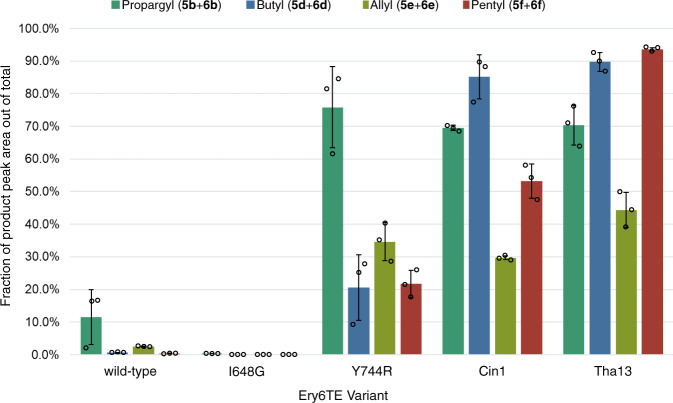
Product distributions catalyzed by motif-swapped Ery6TE variants with two competing substrates. Ery6TE extension unit competition assay with 1.5 mM **1** and 1.5 mM each of either **2b**, **2d**, **2e**, or **2f**. Average percentage of each indicated product out of the total products in each reaction as determined by HR-LC-MS analysis. Error bars are the standard deviation (*n* = 3 biological replicates) of the mean. Individual replicates are shown as circles. Source data are provided as a Source Data file.

Next, to better mimic expected in vivo conditions in an engineered strain^27^, the product distribution catalyzed by the wild-type Ery6TE, I648G, Y744R, and the two motif-swapped chimeras, was determined using high concentrations (1.5 mM) of the native Ery6TE substrate (**1**) in equimolar competition with just one other extender unit (either **2b**, **2d**, **2e**, or **2f**, Fig. 4a). Notably, under these conditions, the wild-type Ery6TE is remarkably stringent. Only ≤ 12% of the products are derived from **2b**, **2d**, **2e**, or **2** **f**, respectively, in each reaction (Fig. 7 and Supplementary Table 5), indicating that the **1**-derived macrolactones are the major products. As expected, Y744R inverted the AT selectivity towards the propargyl products (**5b** + **6b**), but the single mutation could not invert the substrate preference from methyl to any of the other three extender units tested. The I648G mutation proved even more stringent for the native extender **1** under these conditions, with only 0.3% incorporation of **2b** and no incorporation of any of the remaining extenders. In contrast, >50% of the products catalyzed by the Ery6(Tha13)TE and Ery6(Cin1)TE chimeras were the propargyl (**5b** + **6b**), butyl (**5d** + **6d**), and pentyl (**5** **f** + **6** **f**) macrolactones in each competition assay, indicating a complete inversion of substrate selectivity toward each corresponding extender unit over the native methyl substrate **1**. Notably, for Ery6(Tha13)TE, the pentyl keto analog (**6f**) comprised 93.5% of the total products when **2f** was in direct competition with the native extender unit **1**, a ~300-fold increase in substrate selectivity as compared to wild-type Ery6TE. More surprisingly, considering that CinAT1 takes hexylmalonyl-CoA in its native pathway, was the decrease in **2f** incorporation by Ery6(Cin1)TE relative to incorporation of the smaller **2b** and **2d**. As expected from analysis of the distribution of reduced and non-reduced products with **2a**-**2d** and our prior studies with the pikromycin PKS, most of the allyl products were not reduced while none of the pentyl products had been processed by the KR (Supplementary Table 5)^20^.

## Discussion

Engineering PKSs to accept non-native extender units is a necessary step towards diversifying bioactive polyketide core structures. To date, there have been several advances towards introduction of non-natural moieties using native AT promiscuity^22,28^, *trans*-ATs^27,29–31^, full AT swaps^14^, and AT active site mutagenesis^16–20,24^. Despite these advances, our lack of insight into the molecular and structural basis for substrate selectivity has meant targeting the same small number of AT residues without exploring the remainder of the ~430-residue domain or even the entire active site. Engineering selectivity between substrates often differing in only a single carbon unit without a defined binding pocket is a unique protein engineering challenge. Here, this was tackled through modeling the AT’s internal dynamics and exploration of non-traditional residues either individually or as part of structural motifs.

Guided by MD simulations, sequence analysis, and previous mutagenesis data, a total of 13 mutations (spanning 10 residues) out of less than 50 that were screened in vitro, resulted in significant (1.5- to >10-fold) increases in selectivity towards a provided extender. These selectivity-shifting mutations were identified throughout EryAT6’s active site, particularly in the areas proximal to the YASH loop (SSM) or the VDVVQP motif (LSM). Notably, the single mutations did not always simply increase overall substrate promiscuity but shifted selectivity towards specific extenders (e.g., L676V increased butyl incorporation at the expense of propargyl).

Notably, and consistent with previous studies, the MD simulations suggested that the previously tested combination of V742A/Y744R is unstable^16^. This might reflect the approach used to develop these first-generation mutations, whereby mutations were identified by screening small libraries of single mutants, which were then arbitrarily combined. This does not guarantee any additivity or synergy between mutations. Gratifyingly however, the substrate specificity of the AT was dramatically shifted via larger structural motif swaps that targeted the residues highlighted by MD simulations and single site mutagenesis. Harnessing the inter- and intra-motif interactions avoids the issues provoked by full domain swaps by leaving >95% of the AT unchanged^3^. As such, both chimeras, Ery6(Tha13)TE and Ery6(Cin1)TE, retained relatively high overall activities while shifting substrate selectivity almost completely towards larger substrates, as anticipated according to the presumed native preference of each parent AT (e.g., Ery6(Tha13)TE realized a > 300-fold change in pentyl (**1g**) incorporation compared to wild-type Ery6TE).

In combination with the in vitro assay results, the MD simulations of EryAT6 afford the most complete picture of a PKS AT domain to date. The simulations provide a description of the dynamics of the entire domain and the effect(s) of mutations at various residues throughout the active site. Several additional insights were enabled via this comprehensive systematic approach. Examination of the mass spectra of the cyclized products produced by Ery6TE revealed the expected ions corresponding to non-natural incorporation (i.e., **4b**–**e**) but also ions corresponding to the unreduced 10-dML products (i.e., **5b**–**g**, Fig. 4b). This lack of KR tolerance towards the C2 extender position, only briefly reported before^20^, will play a major role in future PKS engineering, especially with larger substrates, as the butyl and pentyl products were not reduced at all. Thus, overcoming the stringent specificity of EryAT6 has revealed a bottleneck that needs to be overcome to capture the full catalytic capabilities of the PKS. An additional challenge that arises is the evolution of ATs capable of recognizing more structurally diverse extenders. While focus needs to be placed on engineering promiscuity of the KR and KS domains, it has been shown here that AT substrate selectivity can be modified using individual and grouped mutations throughout the AT active site, and it is anticipated that the number of implicated residues and incorporated substrates will grow as more are explored. Finally, this combined in silico and mutagenesis approach can be readily expanded to include other AT and substrate combinations and is expected to advance future engineering of the AT domain. This is expected to advance our ability to access designer polyketides and to facilitate structure-function studies in complex natural products.

## Methods

### General information

Materials and reagents were purchased from Sigma Aldrich unless otherwise noted. Isopropyl β-D-thiogalactoside (IPTG) was purchased from Calbiochem. The *E. coli* K207-3 strain was provided by Dr. Adrian Keatinge-Clay at the University of Texas at Austin^32^. Construction of the pET28-Ery6TE plasmid was described previously^16^. Briefly, the pikromycin docking domain fused to the *ery6TE* sequence was cloned using *Nde*I and *Hin*dIII into pET28a. All module sequences are listed in Supplementary Table 7. Primers were purchased from Integrated DNA Technologies. All *holo* proteins were expressed in *E. coli* K207-3 cells containing *sfp*, and all *apo* proteins were expressed in *E. coli* BL21 (DE3) cells. All oligo sequences are listed in Supplementary Table 8.

### Homology models and molecular dynamics

Wild-type homology models for EryAT6 (with different boundaries) were created using the I-TASSER online server^33–35^. I-TASSER was also used to create homology models of EryAT6 with motif swaps. Mutations were introduced into wild-type models with YASARA Structure^36–38^. Molecular graphics and analyses of MD trajectories and PDB snapshots were performed with UCSF Chimera 1.10.1^39,40^.

For the EryAT6 models lacking one or both linkers, MD simulations using the standard YASARA macro md_run were run for 50 ns for each model at 30 °C and pH 7.4 using YASARA Structure software^36,41^ with the AMBER14 force field^37,42,43^. The cell was filled using the TIP3P water model at pH 7.4 and 298 K. An ionic concentration of 0.9% NaCl was used. pK_a_ values were determined for each residue^37^. The proteins were simulated in a periodic cuboid with a 10 Å boundary and an 8 Å PME cutoff value and 5 fs timesteps. Each simulation was repeated with a different random seed and starting velocities to support the reproducibility of observations. Retention of secondary structures was also evaluated (Supplementary Fig. 3).

For all EryAT6 models with both linkers present, MD simulations using the standard YASARA macro md_run were run for 100 ns for each model at 30 °C and pH 7.4 using YASARA Structure software^36,41^ with the AMBER14 force field^37,42,43^. The cell was filled using the TIP3P water model at pH 7.4 and 298 K. An ionic concentration of 0.9% NaCl was used. pK_a_ values were determined for each residue^37^. The proteins were simulated in a periodic cuboid with a 10 Å boundary and an 8 Å PME cutoff value and 2.5 fs timesteps. Each simulation was repeated with a different random seed to support convergence and observations. Where possible, time-averaged structures were used, but examples of snapshots from the simulation trajectories were selected from the second half of the simulations and were used to show representative snapshots as determined by manual inspection of the trajectories. RMSD, RMSF, distances, and other analyses were performed using YASARA Structure for the final 80 ns of each simulation, with 20 ns allowed as an equilibration period^36^.

### Expression and purification of wild-type and mutant Ery6TE

Cells were grown in 300 mL of LB media with 50 μg mL^−1^ kanamycin at 37 °C and 250 rpm until OD_600_ reached ~0.6, and then the culture was induced with 1 mM IPTG and allowed to express at 18 °C for 20 h. Proteins were purified on a nickel column with a Bio-Rad BioLogic LP FPLC (Wash buffer: 50 mM sodium phosphate, pH 7.2, 300 mM NaCl, 20 mM imidazole, and EDTA-free protease inhibitor cocktail (Roche); Elution buffer: 50 mM sodium phosphate, pH 7.2, 300 mM NaCl, 200 mM imidazole, and EDTA-free protease inhibitor cocktail), concentrated with a 10 kDa MWCO filter (EMD Millipore), and stored as single-use aliquots in module storage buffer (100 mM sodium phosphate, pH 7.4, 1 mM EDTA, 1 mM tris(2-carboxyethyl)phosphine (TCEP), 20% v/v glycerol, 0.1 μL Benzonase (NEB), and EDTA-free protease inhibitor cocktail) at −80 °C.

### MatB reactions and acyl-CoA preparation

Wild-type MatB and the mutant MatB T207G/M306I were purified and 8 mM stocks of malonyl-CoA analogs (**1**, **2a**–**f**, Supplementary Fig. 8) were set up as previously disclosed^44^ and described in the Supplementary Methods. Stocks were normalized based on completion percentage before addition to pentaketide assays.

### Ery6TE lysate preparation

Modules (wild-type and mutant Ery6 and Ery6TE) were expressed overnight at 18 °C in 300 mL cultures in LB media with the appropriate antibiotics. Protein production was induced with 1 mM IPTG at OD_600_ of ~0.6. After overnight expression, the culture was centrifuged at 5000 × *g* for 20 min, and the supernatant was discarded. The cells were resuspended in 1 mL module storage buffer and sonicated using 51% amplitude, 10 s on, 20 s off for 10 min. After sonication, the lysed cells were centrifuged at 52,400 × *g* for 1 h. The lysates were aliquoted and stored at −80 °C. Protein expression was verified by sodium dodecyl sulphate–polyacrylamide gel electrophoresis (SDS-PAGE). Protein quantification was carried out using the Bradford Protein Assay Kit from Bio-Rad.

### Ery6TE pentaketide assay

The pentaketide assay was set up with a total volume of 80 μL in 100 mM sodium phosphate, pH 7.0, and 2 mM MgCl_2_. The reaction conditions included 1 mM TP-pentaketide (**3**), 1.75 mM of each competing malonyl-CoA from MatB reactions (**1**, **2a**–**f**), an NADPH regeneration system (5 mM glucose-6-phosphate, 500 μM NADP + , and 0.008 U mL^−1^ glucose-6-phosphate dehydrogenase), and lysate containing the module. Module concentrations were calculated by Bradford assay and SDS-PAGE gel analysis, and results were normalized based on protein content for each reaction. Negative controls were run with apo modules lacking the phosphopantetheine modifications on the ACP domains (using lysates without Sfp). Reactions proceeded at room temperature for 16 h and were quenched with an equal volume of −20 °C methanol. After quenching, all reactions were centrifuged at 10,000 × *g* three times for 3 h total, and the supernatant was filtered through a nylon 0.2 μm filter. Analysis was carried out on a high-resolution mass spectrometer (ThermoFisher Scientific Exactive Plus MS, a benchtop full-scan Orbitrap™ mass spectrometer) using Heated Electrospray Ionization (HESI). The sample was analyzed via LC-MS injection into the mass spectrometer at a flow rate of 225 µL min^−1^. The mobile phase B was acetonitrile with 0.1% formic acid and mobile phase A was water with 0.1% formic acid (see Supplementary Table 9 for gradient and scan parameters). The mass spectrometer was operated in positive ion mode. The LC column was a Thermo Hypersil Gold 50  × 2.1 mm, 1.9 µm particle size. This assay produces 10-dML and narbonolide products that can be detected as their [M + H] + , [M + H-H_2_O]^+^, and [M + Na]^+^ ions and keto-10-dML products that can be detected as their [M + H]^+^ and [M + Na]^+^ ions. Extracted ions for each listed ion were summed for comparison purposes. Peak areas for extracted ions were used to calculate product peak areas. Every assay was repeated three distinct times, unless otherwise stated. For retention times, calculated masses, observed masses, representative extracted ion counts, and representative chromatograms, see Supplementary Tables 2, 3, 5, and Supplementary Figs. 10 and 11.

### Reporting summary

Further information on research design is available in the Nature Research Reporting Summary linked to this article.

## Supplementary information

Supplementary Information

Reporting Summary

## Data Availability

The data that support the in vitro findings (e.g., enzyme assays) of this study are available within the paper or its supplementary information. The data that support the computational findings (e.g., MD simulations) are available from the corresponding author upon reasonable request. Source data are provided with this paper.

## Source data

Source Data
