# Supplementary material for: Computationally-guided exchange of substrate selectivity motifs in a modular polyketide synthase acyltransferase
Source: Nat Commun. 2021 Apr 13;12:2193. doi: 10.1038/s41467-021-22497-2 (PMC8044089; doi:10.1038/s41467-021-22497-2)
Supplement: Supplementary file 1 — Supplementary Information [file 41467_2021_22497_MOESM1_ESM.pdf]

Supplementary Information

Computationally-guided exchange of substrate selectivity motifs in a modular polyketide synthase acyltransferase

Edward Kalkreuter[1,2,3], Kyle S Bingham[1,4], Aaron M Keeler[1], Andrew N Lowell[5,7], David H Sherman[5,6], and Gavin J Williams[1,2]*

[1] Department of Chemistry, NC State University, Raleigh, North Carolina 27695, United States

[2] Comparative Medicine Institute, NC State University, Raleigh, North Carolina 27695, United States

[3] Present address: Department of Chemistry, The Scripps Research Institute, Jupiter, Florida 33458, United States

[4] Present address: UNC Chapel Hill School of Medicine, Chapel Hill, North Carolina 27516, United States

[5] Life Sciences Institute, Department of Medicinal Chemistry, University of Michigan, Ann Arbor, Michigan 48109, United States

[6] Department of Chemistry, Department of Microbiology & Immunology, University of Michigan, Ann Arbor, Michigan 48109, United States

[7] Present address: Department of Chemistry, Virginia Tech, Blacksburg, Virginia 24061, United States

* Corresponding author, gjwillia@ncsu.edu

Table of contents

Supplementary Tables

Supplementary Table 1. Wild-type EryAT6 amino acid sequences for MD simulations.

Supplementary Table 2. High-Resolution LC-MS retention times, calculated masses, and observed masses for DEBS PKS-catalyzed reaction products.

Supplementary Table 3. High-resolution LC-MS peak areas for PKS-catalyzed reaction products with five extenders.

Supplementary Table 4. Mutations in Ery6TE that did not result in a shift from the wild-type product distribution.

Supplementary Table 5. High-resolution LC-MS peak areas for PKS-catalyzed reaction products with two extenders.

Supplementary Table 6. Statistics for high-resolution LC-MS peak areas for PKS-catalyzed reaction products with two extenders.

Supplementary Table 7. DEBS PKS DNA FASTA sequences.

Supplementary Table 8. Primers for DEBS PKS construction and mutagenesis.

Supplementary Table 9. High-resolution LC-MS parameters and gradient.

Supplementary Figures

Supplementary Figure 1. Conserved motifs of PKS acyltransferases.
Supplementary Figure 2. Evaluation of EryAT6 models.
Supplementary Figure 3. Per-residue protein secondary structure of EryAT6 models.
Supplementary Figure 4. RMSD of EryAT6 MD simulations.
Supplementary Figure 5. Time-averaged structures of EryAT6 variants.
Supplementary Figure 6. RMSF plots for EryAT6 simulations.
Supplementary Figure 7. Distance plots for EryAT6 simulations.
Supplementary Figure 8. Representative HPLC traces for MatB-synthesized malonyl-CoAs.
Supplementary Figure 9. Motif sequences from natural acyltransferases.
Supplementary Figure 10. Representative mass spectra of 10-deoxymethynolide B compounds from lysate module reactions.
Supplementary Figure 11. Representative mass spectra of keto-10-deoxymethynolide B compounds from lysate module reactions.

Supplementary Methods

Site-directed mutagenesis of Ery6TE
Construction of Ery6TE motif chimeras
Expression and purification of wild-type and mutant MatB
Synthesis of acyl-CoAs by MatB

Supplementary References

Supplementary Tables

Supplemental Table 1. Wild-type EryAT6 amino acid sequences for MD simulations. Linkers are in bold.

EryAT6 Construct	Amino Acid FASTA Sequences for MD Simulations
**Wild-Type with Both Linkers**	**AEPPEPEPLPEPGPVGVLAAANSVPVLLSARTETALAAQARLLESAVDDSVPLTA**<br>**LASALATGRAHLPRRAALLAGDHEQLRGQLRAVAEGVAAPGATTGTASAGGVV**<br>**F**VFPGQGAQWEGMARGLLSVPVFAESIAECDAVLSEVAGFSASEVLEQRPDAPSL<br>ERVDVVQPVLFSVMVSLARLWGACGVSPSAVIGHSQGEIAAAVVAGVLSLEDGVR<br>VVALRAKALRALAGKGGMVSLAAPGERARALIAPWEDRISVAAVNSPSSVVVSGD<br>PEALAELVARCEDEGVRAKTLPVDYASHSRHVEEIRETILADLDGISARRAAIPLYST<br>LHGERRDGADMGPRYWYDNLRSQVRFDEAVSAAVADGHATFVEMSPHPVLTAA<br>VQE**IAADAVAIGSLHRDTAEEHLIAELARAHVHGVAVDWRNVFPAA**
**Wild-Type with N-Terminal Linker**	**AEPPEPEPLPEPGPVGVLAAANSVPVLLSARTETALAAQARLLESAVDDSVPLTA**<br>**LASALATGRAHLPRRAALLAGDHEQLRGQLRAVAEGVAAPGATTGTASAGGVV**<br>**F**VFPGQGAQWEGMARGLLSVPVFAESIAECDAVLSEVAGFSASEVLEQRPDAPSL<br>ERVDVVQPVLFSVMVSLARLWGACGVSPSAVIGHSQGEIAAAVVAGVLSLEDGVR<br>VVALRAKALRALAGKGGMVSLAAPGERARALIAPWEDRISVAAVNSPSSVVVSGD<br>PEALAELVARCEDEGVRAKTLPVDYASHSRHVEEIRETILADLDGISARRAAIPLYST<br>LHGERRDGADMGPRYWYDNLRSQVRFDEAVSAAVADGHATFVEMSPHPVLTAA<br>VQE
**Wild-Type with No Linkers**	VFPGQGAQWEGMARGLLSVPVFAESIAECDAVLSEVAGFSASEVLEQRPDAPSLE<br>RVDVVQPVLFSVMVSLARLWGACGVSPSAVIGHSQGEIAAAVVAGVLSLEDGVRV<br>VALRAKALRALAGKGGMVSLAAPGERARALIAPWEDRISVAAVNSPSSVVVSGDP<br>EALAELVARCEDEGVRAKTLPVDYASHSRHVEEIRETILADLDGISARRAAIPLYSTL<br>HGERRDGADMGPRYWYDNLRSQVRFDEAVSAAVADGHATFVEMSPHPVLTAAV<br>QE

Supplemental Table 2. High-Resolution LC-MS retention times, calculated masses, and observed masses for DEBS PKS-catalyzed reaction products. N.D. = Not Detected.

Compound &	Retention Time (min)	Calculated Mass ($[M-H_2O+H]^+$)	Observed Mass ($[M-H_2O+H]^+$)	Calculated Mass ($[M+H]^+$)	Observed Mass ($[M+H]^+$)	Calculated Mass ($[M+Na]^+$)	Observed Mass ($[M+Na]^+$)
**4**	8.03	279.1955	279.1962	297.2060	297.2069	319.1880	319.1904
**5a**	9.68	293.2111	293.2119	311.2217	311.2227	333.2036	333.2044
**5b**	8.96	303.1955	303.1963	321.2060	321.2068	343.1880	343.1890
**5c**	10.63	307.2268	307.2274	325.2373	325.2378	347.2193	347.2198
**5d**	N.D.	321.2424	N.D.	339.2530	N.D.	361.2349	N.D.
**5e**	10.12	305.2111	305.2105	323.2217	323.2201	345.2036	345.2035
**5f**	N.D.	335.2581	N.D.	353.2686	N.D.	375.2506	N.D.
**6a**	12.39	--	--	309.2060	309.2067	331.1880	331.1886
**6b**	11.80	--	--	319.1904	319.1911	341.1723	341.1732
**6c**	11.79	--	--	323.2217	323.2224	345.2036	345.2044
**6d**	14.56	--	--	337.2373	337.2380	359.2193	359.2200
**6e**	12.67	--	--	321.2060	321.2055	343.1880	343.1876
**6f**	15.07	--	--	351.2530	351.2525	373.2349	373.2345

Supplemental Table 3. High-resolution LC-MS peak areas for PKS-catalyzed reaction products with five extenders. Extracted ion count peak areas from one replicate are shown for each reaction condition, prior to correction with enzyme concentration. N.D. = Not Detected.

Ery6TE Variant	Representative EIC Peak Areas				
	**4**	**5a**	**6a**	**5b**	**6b**
WT	211,060,257	8,350,707	3,201,023	60,428,975	33,336,885
V742A	1,074,530,536	74,181,724	8,289,132	621,019,227	62,604,070
Y744R	201,560,426	18,273,398	10,450,848	425,370,308	221,736,187
L673G	412,033,607	11,485,854	3,946,441	96,211,067	47,618,973
A672S	918,117,379	17,567,468	16,886,399	174,246,461	190,537,319
L676V	913,134,214	20,437,965	21,610,476	202,357,895	254,127,057
G681A	258,612,993	7,211,911	4,379,076	42,211,949	38,450,516
D613E	1,112,681,260	25,901,052	23,610,928	246,571,818	263,851,451
R674A	1,137,937,000	22,945,009	25,007,975	178,486,582	202,512,483
H643S	244,645,247	12,743,255	8,023,759	70,681,023	47,042,985
H643F	215,279,432	1,629,754	960,271	2,131,336	2,185,209
I648G	365,230,755	869,632	475,403	1,770,386	1,262,385
L797E	565,835,459	782,613	2,352,056	829,163	1,769,043
Cin1	1,122,551	14,392	29,947	1,093,275	2,750,716
Tha13	29,754,912	1,565,605	928,229	31,038,395	17,687,295

Ery6TE Variant	Representative EIC Peak Areas			
	**5c**	**6c**	**5d**	**6d**
WT	11,975,213	22,923,302	N.D.	3,401,711
V742A	41,645,025	4,688,419	N.D.	32,702,810
Y744R	87,834,327	60,330,553	N.D.	110,340,604
L673G	12,692,412	27,903,903	N.D.	15,313,652
A672S	7,799,155	195,172,467	N.D.	18,325,167
L676V	2,065,221	161,120,479	N.D.	28,957,619
G681A	13,320,019	63,892,775	N.D.	6,301,166
D613E	20,124,173	244,569,998	N.D.	37,219,146
R674A	16,724,087	256,290,037	N.D.	19,347,354
H643S	25,063,933	35,489,999	N.D.	18,784,167
H643F	74,197	3,727,611	N.D.	589,128
I648G	595,456	6,006,129	N.D.	1,376,049
L797E	128,196	1,623,863	N.D.	1,573,907
Cin1	39,917	439,077	N.D.	1,402,533
Tha13	22,492,383	39,231,371	N.D.	123,779,145

Supplemental Table 4. Mutations in Ery6TE that did not result in a shift from the wild-type product distribution. All mutants that were constructed and tested in vitro with **1** and non-native extenders but showed no difference in product distribution (other than total activity) from wild-type Ery6TE were excluded from the main text but are included in the following table.

Ery6TE Mutants Indistinguishable from Wild-Type		
A596L	A672S	L740V
E598D	R674A	P741Δ
V612D	A675L	P741G
E647D	G689A	P790A
G662A	D701G	L797S
A670T	A707G	
A670V	S717A	

Supplemental Table 5. High-resolution LC-MS peak areas for PKS-catalyzed reaction products with two extenders. Extracted ion count peak areas from the three replicates are shown for each reaction condition, prior to correction with enzyme concentration. N.D. = Not Detected.

		EIC Peak Areas				
**Substrates**	**Product**	**WT**	**I648G**	**Y744R**	**Cin1**	**Tha13**
1+2b	4	532,578,041	799,908,718	78,684,251	29,848,810	26,068,751
	5b	31,090,929	2,583,102	320,918,411	20,904,574	45,737,083
	6b	75,186,842	N.D.	109,088,198	44,368,738	37,497,560
	4	716,257,624	1,281,730,797	288,363,018	27,967,173	110,869,247
	5b	10,491,816	3,456,956	93,514,897	20,078,581	129,467,348
	6b	3363144	N.D.	367,954,412	45,478,751	66,654,955
	4	663,536,505	851,319,123	454,754,654	25,447,589	27,668,082
	5b	90,668,644	3,371,564	1,416,351,093	14,412,667	18,014,853
	6b	37,810,814	N.D.	563,471,339	44,512,521	49,507,603
1+2d	4	1,241,364,749	650,111,454	230,399,192	478,245	29,616,734
	5d	N.D.	N.D.	N.D.	N.D.	N.D.
	6d	6,953,819	N.D.	88,178,801	4,152,337	193,602,896
	4	442,846,997	701,947,321	40,688,455	18,686,181	13,798,643
	5d	N.D.	N.D.	N.D.	N.D.	N.D.
	6d	3,252,033	N.D.	13,599,517	64,279,072	172,045,949
	4	1,109,471,085	1,318,378,357	353,628,391	17,770,595	56,310,379
	5d	N.D.	N.D.	N.D.	N.D.	N.D.
	6d	6,247,017	N.D.	35,401,742	134,608,436	499,303,732
1+2e	4	1,263,268,254	1,467,946,423	122,464,111	45,392,299	43,610,053
	5e	7,647,855	N.D.	28,872,748	964,634	10,197,042
	6e	21,784,302	N.D.	53,689,472	17,802,789	33,024,254
	4	1,347,621,460	1,166,990,061	231,683,367	41,470,130	54,256,708
	5e	8,642,746	N.D.	31,211,506	5,053,195	12,186,663
	6e	27,600,123	N.D.	61,477,390	13,093,086	22,590,082
	4	749,218,220	787,105,102	395,020,747	40,718,563	60,618,920
	5e	3,627,008	N.D.	55,760,678	6,361,402	18,086,700
	6e	14,433,977	N.D.	157,017,607	10,680,958	30,154,791
1+2f	4	1,144,743,869	1,115,568,669	622,633,725	29,298,295	30,377,170
	5f	N.D.	N.D.	N.D.	N.D.	N.D.
	6f	1,583,892	N.D.	217,518,820	26,459,590	473,073,518
	4	905,943,636	1,217,656,413	98,396,869	33,943,300	20,218,413
	5f	N.D.	N.D.	N.D.	N.D.	N.D.
	6f	3,663,759	N.D.	27,012,724	40,092,569	271,527,843
	4	1,153,814,036	813,348,137	880,467,719	34,365,774	39,255,832
	5f	N.D.	N.D.	N.D.	N.D.	N.D.
	6f	4,307,982	N.D.	187,905,304	47,369,923	602,913,839

Supplemental Table 6. Statistics for high-resolution LC-MS peak areas for PKS-catalyzed reaction products with two extenders. Statistics of the extracted ion count peak areas from **Table S5** are shown for each reaction condition. Results also shown in **Figure 7**. N.D. = Not Detected.

		Average Peak Area Distribution					Standard Deviation of Peak Area Distribution				
**Substrates**	**Product**	**WT**	**I648G**	**Y744R**	**Cin1**	**Tha13**	**WT**	**I648G**	**Y744R**	**Cin1**	**Tha13**
**1 + 2b**	**4**	88.4%	99.7%	24.2%	30.5%	29.7%	8.4%	0.1%	12.4%	0.8%	6.2%
	**5b + 6b**	11.6%	0.3%	75.8%	69.5%	70.3%	8.4%	0.1%	12.4%	0.8%	6.2%
**1 + 2d**	**4**	99.4%	100.0%	79.4%	14.8%	10.3%	0.1%	0.0%	10.1%	6.7%	2.9%
	**5d + 6d**	0.6%	0.0%	20.6%	85.2%	89.7%	0.1%	0.0%	10.1%	6.7%	2.9%
**1 + 2e**	**4**	97.6%	100.0%	65.4%	70.3%	55.6%	0.2%	0.0%	5.9%	0.6%	5.4%
	**5e + 6e**	2.4%	0.0%	34.6%	29.7%	44.4%	0.2%	0.0%	5.9%	0.6%	5.4%
**1 + 2f**	**4**	99.7%	100.0%	78.3%	46.8%	6.4%	0.1%	0.0%	4.2%	5.3%	0.5%
	**5f + 6f**	0.3%	0.0%	21.7%	53.2%	93.6%	0.1%	0.0%	4.2%	5.3%	0.5%

Supplemental Table 7. DEBS PKS DNA FASTA sequences. AT domains in modules are in bold. Pik docking domain is italicized.

Construct	Nucleotide Sequences
**ThaAT13 Motifs**	GACCCAATACGCGCCGTCGCTGGAGCGTCTGGATGTGAATCAGCCGGTTCTGTTTAGT<br>GTTATGGTGAGTCTGGCCCGTCTGTGGGGTGCCTGTGGTGTGAGTCCGAGTGCCGTTA<br>TTGGCCATAGCCAGGGTGAAATTGCAGCAGCAGTTGTTGCAGGCGTGCTGAGTCTGGA<br>AGATGGTGTGCGCGTGGTTGCACTGCGTGCCAAAGCACTGCGTGCGCTGGCAGGCAAA<br>GGTGGCATGGTTAGCCTGGCCGCACCGGGCGAACGCGCTAGAGCTCTGATTGCCCCGT<br>GGGAAGATCGTATTAGCGTTGCCGCAGTTAATAGTCCGAGTAGCGTGGTGGTGAGTGG<br>TGACCCGGAAGCCCTGGCCGAACTGGTGGCACGTTGTGAAGATGAAGGTGTTCGCGCA<br>AAAGTTCTGCCGGGTGCCGATGCCGCAGGCCACTCCCGCCACGTCGAGACCCAAT
**CinAT1 Motifs**	ACGCGCCGTCGCTGGAGGAAGGCGATATTCAGCAGCCGGTTCTGTTTAGTGTGATGGT<br>TAGTCTGGCACGCCTGTGGGGCGCCTGCGGTGTTAGTCCGAGTGCAGTTATTGGCCAT<br>AGCCAGGGCGAAATTGCAGCCGCCGTTGTTGCAGGCGTTCTGAGCCTGGAAGATGGCG<br>TTCGTGTTGTGGCCCTGCGCGCCAAAGCACTGCGCGCACTGGCAGGCAAAGGTGGTAT<br>GGTGAGTCTGGCAGCACCGGGTGAACGCGCCCGTGCTCTGATTGCACCGTGGGAAGAT<br>CGCATTAGCGTGGCCGCAGTGAATAGCCCGAGCAGCGTTGTTGTTAGTGGCGATCCGG<br>AAGCCCTGGCAGAACTGGTGGCCCGTTGCGAAGATGAAGGCGTTCGTGCCAAAGCCCT<br>GCGCGTGGAACGCGCAGGCCACTCCCGCCACGTCGA
**Ery6 WT**	ATGGTCGGCGCAGCAGAGGCGGAGCAAGCCCCGGCGCTCGTGCGCGAGGTGCCGAAGG<br>ATGCCGACGACCCGATCGCGATCGTCGGCATGGCCTGCCGCTTCCCCGGCGGCGTGCA<br>CAACCCCGGTGAGCTGTGGGAGTTCATCGTCGGCGGCGGAGACGCCGTGACGGAGATG<br>CCCACCGACCGCGGCTGGGACCTCGACGCGCTGTTCGACCCCGACCCGCAGCGCCACG<br>GAACCAGCTACTCGCGACACGGCGCGTTCCTCGACGGGGCCGCCGACTTCGACGCGGC<br>GTTCTTCGGGATCTCGCCGCGCGAGGCGCTGGCGATGGACCCGCAGCAGCGCCAGGTC<br>CTGGAAACGACGTGGGAGCTGTTCGAGAACGCCGGCATCGACCCGCACTCGCTGCGGG<br>GCAGCGACACCGGCGTCTTCCTCGGCGCCGCGTACCAGGGCTACGGCCAGGACGCGGT<br>GGTGCCCGAGGACAGCGAGGGCTACCTGCTCACCGGCAACTCCTCCGCCGTGGTGTCC<br>GGCCGGGTCGCCTACGTGCTGGGGCTGGAAGGCCCCGCGGTCACGGTGGACACGGCGT<br>GTTCGTCGTCGTTGGTGGCCTTGCATTCGGCGTGTGGGTCGTTGCGTGACGGTGACTG<br>CGGTCTTGCGGTGGCCGGTGGTGTGTCGGTGATGGCGGGCCCGGAGGTGTTCACCGAG<br>TTCTCCCGCCAGGGCGGCTTGGCCGTGGACGGGCGCTGCAAGGCGTTCTCCGCGGAGG<br>CCGACGGCTTCGGTTTCGCCGAGGGCGTCGCGGTGGTCCTGCTCCAGCGGTTGTCCGA<br>CGCCCGCAGGGCGGGTCGCCAGGTGCTCGGCGTGGTCGCGGGCTCGGCGATCAACCAG<br>GACGGCGCGAGCAACGGTCTCGCGGCGCCGAGCGGCGTCGCCCAGCAGCGCGTGATCC<br>GCAAGGCGTGGGCGCGTGCGGGGATCACGGGCGCGGATGTGGCCGTGGTGGAGGCGCA<br>TGGGACCGGTACGCGGCTGGGCGATCCGGTGGAGGCGTCGGCGTTGCTGGCTACTTAC<br>GGCAAGTCGCGCGGGTCGTCGGGCCCGGTGCTGCTGGGTTCGGTGAAGTCGAACATCG<br>GTCACGCGCAGGCGGCCGCGGGTGTCGCGGGCGTGATCAAGGTGGTCCTGGGGTTGAA<br>CCGCGGCCTGGTGCCGCCGATGCTCTGCCGCGGCGAGCGGTCGCCGCTGATCGAATGG<br>TCCTCGGGTGGTGTGGAACTTGCCGAGGCCGTGAGCCCGTGGCCTCCGGCCGCGGACG<br>GGGTGCGCCGGGCCGGTGTGTCGGCGTTCGGGGTGAGC**GGGACGAACGCGCACGTGAT**<br>**CATCGCCGAGCCCCCGGAGCCCGAGCCGCTGCCGGAACCCGGACCGGTGGGCGTGCTG**<br>**GCCGCTGCGAACTCGGTGCCCGTACTGCTGTCGGCCAGGACCGAGACCGCGTTGGCAG**<br>**CGCAGGCGCGGCTCCTGGAGTCCGCAGTGGACGACTCGGTTCCGTTGACGGCATTGGC**<br>**TTCCGCGCTGGCCACCGGACGCGCCCACCTGCCGCGTCGTGCGGCGTTGCTGGCAGGC**<br>**GACCACGAACAGCTCCGCGGGCAGTTGCGAGCGGTCGCCGAGGGCGTTGCGGCTCCCG**<br>**GTGCCACCACCGGAACCGCCTCCGCCGGCGGCGTGGTTTTCGTCTTCCCAGGTCAGGG**<br>**TGCTCAGTGGGAGGGCATGGCCCGGGGCTTGCTCTCGGTCCCCGTCTTCGCCGAGTCG**

	**ATCGCCGAGTGCGATGCGGTGTTGTCGGAGGTGGCCGGGTTCTCGGCCTCCGAAGTGC**<br>**TGGAGCAGCGTCCGGACGCGCCGTCGCTGGAGCGGGTCGACGTCGTACAGCCGGTGTT**<br>**GTTCTCCGTGATGGTGTCGCTGGCGCGGCTGTGGGGCGCTTGCGGAGTCAGCCCCTCG**<br>**GCCGTCATCGGCCATTCGCAGGGCGAGATCGCCGCCGCGGTGGTGGCCGGGGTGTTGT**<br>**CGCTGGAGGACGGCGTGCGCGTCGTGGCCCTGCGCGCGAAGGCGTTGCGTGCGCTGGC**<br>**GGGCAAGGGCGGCATGGTCTCGTTGGCGGCTCCCGGTGAACGCGCCCGCGCGCTGATC**<br>**GCACCGTGGGAGGACCGGATCTCCGTCGCGGCGGTCAACTCCCCGTCCTCGGTCGTGG**<br>**TCTCCGGCGATCCGGAGGCGCTGGCCGAACTCGTCGCACGTTGCGAGGACGAGGGCGT**<br>**GCGCGCCAAGACGCTCCCGGTGGACTACGCCTCGCACTCCCGCCACGTCGAGGAGATC**<br>**CGCGAGACGATCCTCGCCGACCTCGACGGCATCTCCGCGCGGCGTGCCGCCATCCCGC**<br>**TCTACTCCACGCTGCACGGCGAACGGCGCGACGGCGCCGACATGGGTCCGCGGTACTG**<br>**GTACGACAACCTGCGCTCCCAGGTGCGCTTCGACGAGGCGGTCTCGGCCGCCGTCGCC**<br>**GACGGTCACGCCACCTTCGTCGAGATGAGCCCGCACCCGGTGCTCACCGCGGCGGTGC**<br>**AGGAGATCGCCGCGGACGCCGTGGCCATCGGGTCGCTGCACCGCGACACCGCGGAGGA**<br>**GCACCTGATCGCCGAGCTCGCCCGGGCGCACGTGCACGGCGTGGCCGTGGACTGGCGG**<br>**AACGTCTTCCCGGCGGCACCTCCGGTGGCG**CTGCCCAACTACCCGTTCGAGCCCCAGC<br>GGTACTGGCTCGCGCCGGAGGTGTCCGACCAGCTCGCCGACAGCCGCTACCGCGTCGA<br>CTGGCGACCGCTGGCCACCACGCCGGTGGACCTGGAAGGCGGCTTCCTGGTCCACGGG<br>TCCGCACCGGAGTCGCTGACCAGCGCAGTCGAGAAGGCCGGAGGCCGCGTCGTGCCGG<br>TCGCCTCGGCCGACCGCGAAGCGCTCGCGGCGGCCCTGCGGGAGGTGCCGGGCGAGGT<br>CGCCGGCGTGCTCTCGGTCCACACCGGCGCCGCAACGCACCTCGCCCTGCACCAGTCG<br>CTGGGTGAGGCCGGCGTGCGGGCCCCGCTCTGGCTGGTCACCAGCCGAGCGGTCGCGC<br>TCGGGGAGTCCGAGCCGGTCGATCCCGAGCAGGCGATGGTGTGGGGTCTCGGGCGCGT<br>CATGGGCCTGGAGACCCCGGAACGGTGGGGCGGTCTGGTGGACCTGCCCGCCGAACCC<br>GCGCCGGGGGACGGCGAGGCGTTCGTCGCCTGCCTCGGCGCGGACGGCCACGAGGACC<br>AGGTCGCGATCCGTGACCACGCCCGCTACGGCCGCCGCCTCGTCCGCGCCCCGCTGGG<br>CACCCGCGAGTCGAGCTGGGAGCCGGCGGGCACGGCGCTGGTCACCGGCGGCACCGGT<br>GCGCTCGGCGGCCACGTCGCCCGCCACCTCGCCAGGTGCGGGGTGGAGGACCTGGTGC<br>TGGTCAGCAGGCGCGGCGTCGACGCTCCCGGCGCGGCCGAGCTGGAAGCCGAACTGGT<br>CGCCCTCGGCGCGAAGACGACCATCACCGCCTGCGACGTGGCCGACCGCGAGCAGCTC<br>TCCAAGCTGCTGGAAGAACTGCGCGGGCAGGGACGTCCGGTGCGGACCGTCGTGCACA<br>CCGCCGGGGTGCCCGAATCGAGGCCGCTGCACGAGATCGGCGAGCTGGAGTCGGTCTG<br>CGCGGCGAAGGTGACCGGGGCCCGGCTGCTCGACGAGCTGTGCCCGGACGCCGAGACC<br>TTCGTCCTGTTCTCGTCCGGAGCGGGGGTGTGGGGCAGTGCGAACCTCGGCGCCTACT<br>CCGCGGCCAACGCCTACCTCGACGCGCTGGCCCACCGCCGCCGTGCGGAAGGCCGTGC<br>GGCGACGTCCGTCGCGTGGGGCGCCTGGGCGGGCGAGGGCATGGCCACCGGCGACCTC<br>GAGGGGCTCACCCGGCGCGGCCTGCGCCCGATGGCGCCCGAGCGCGCGATCCGCGCGC<br>TGCACCAGGCGCTGGACAACGGCGACACGTGCGTTTCGATCGCCGACGTCGACTGGGA<br>GCGCTTCGCGGTCGGCTTCACCGCCGCCCGGCCGCGTCCGCTGCTGGACGAGCTCGTC<br>ACGCCGGCGGTGGGGGCCGTCCCCGCGGTGCAGGCGGCCCCGGCGCGGGAGATGACGT<br>CGCAGGAGTTGCTGGAGTTCACGCACTCGCACGTCGCGGCGATCCTCGGGCATTCCAG<br>CCCGGACGCGGTCGGGCAGGACCAGCCGTTCACCGAGCTCGGCTTCGACTCGCTGACC<br>GCGGTCGGGCTGCGCAACCAGCTCCAGCAGGCCACCGGGCTCGCGCTGCCCGCGACCC<br>TGGTGTTCGAGCACCCCACGGTCCGCAGGTTGGCCGACCACATAGGACAGCAGCTCTG<br>A
**PDDEry6TE WT**	*ATGACGAGTTCCAACGAACAGTTGGTGGACGCTCTGCGCGCCTCTCTCAAGGAGAACG*<br>*AAGAACTCCGGAAAGAGAGCCGTCGCCGGGCCGACCGTCGGCAGGAGGAG*ATCGCGAT<br>CGTCGGCATGGCCTGCCGCTTCCCCGGCGGCGTGCACAACCCCGGTGAGCTGTGGGAG<br>TTCATCGTCGGCGGCGGAGACGCCGTGACGGAGATGCCCACCGACCGCGGCTGGGACC<br>TCGACGCGCTGTTCGACCCCGACCCGCAGCGCCACGGAACCAGCTACTCGCGACACGG

	CGCGTTCCTCGACGGGGCCGCCGACTTCGACGCGGCGTTCTTCGGGATCTCGCCGCGC<br>GAGGCGCTGGCGATGGACCCGCAGCAGCGCCAGGTCCTGGAAACGACGTGGGAGCTGT<br>TCGAGAACGCCGGCATCGACCCGCACTCGCTGCGGGGCAGCGACACCGGCGTCTTCCT<br>CGGCGCCGCGTACCAGGGCTACGGCCAGGACGCGGTGGTGCCCGAGGACAGCGAGGGC<br>TACCTGCTCACCGGCAACTCCTCCGCCGTGGTGTCCGGCCGGGTCGCCTACGTGCTGG<br>GGCTGGAAGGCCCCGCGGTCACGGTGGACACGGCGTGTTCGTCGTCGTTGGTGGCCTT<br>GCATTCGGCGTGTGGGTCGTTGCGTGACGGTGACTGCGGTCTTGCGGTGGCCGGTGGT<br>GTGTCGGTGATGGCGGGCCCGGAGGTGTTCACCGAGTTCTCCCGCCAGGGCGGCTTGG<br>CCGTGGACGGGCGCTGCAAGGCGTTCTCCGCGGAGGCCGACGGCTTCGGTTTCGCCGA<br>GGGCGTCGCGGTGGTCCTGCTCCAGCGGTTGTCCGACGCCCGCAGGGCGGGTCGCCAG<br>GTGCTCGGCGTGGTCGCGGGCTCGGCGATCAACCAGGACGGCGCGAGCAACGGTCTCG<br>CGGCGCCGAGCGGCGTCGCCCAGCAGCGCGTGATCCGCAAGGCGTGGGCGCGTGCGGG<br>GATCACGGGCGCGGATGTGGCCGTGGTGGAGGCGCATGGGACCGGTACGCGGCTGGGC<br>GATCCGGTGGAGGCGTCGGCGTTGCTGGCTACTTACGGCAAGTCGCGCGGGTCGTCGG<br>GCCCGGTGCTGCTGGGTTCGGTGAAGTCGAACATCGGTCACGCGCAGGCGGCCGCGGG<br>TGTCGCGGGCGTGATCAAGGTGGTCCTGGGGTTGAACCGCGGCCTGGTGCCGCCGATG<br>CTCTGCCGCGGCGAGCGGTCGCCGCTGATCGAATGGTCCTCGGGTGGTGTGGAACTTG<br>CCGAGGCCGTGAGCCCGTGGCCTCCGGCCGCGGACGGGGTGCGCCGGGCCGGTGTGTC<br>GGCGTTCGGGGTGAGC**GGGACGAACGCGCACGTGATCATCGCCGAGCCCCCGGAGCCC**<br>**GAGCCGCTGCCGGAACCCGGACCGGTGGGCGTGCTGGCCGCTGCGAACTCGGTGCCCG**<br>**TACTGCTGTCGGCCAGGACCGAGACCGCGTTGGCAGCGCAGGCGCGGCTCCTGGAGTC**<br>**CGCAGTGGACGACTCGGTTCCGTTGACGGCATTGGCTTCCGCGCTGGCCACCGGACGC**<br>**GCCCACCTGCCGCGTCGTGCGGCGTTGCTGGCAGGCGACCACGAACAGCTCCGCGGGC**<br>**AGTTGCGAGCGGTCGCCGAGGGCGTTGCGGCTCCCGGTGCCACCACCGGAACCGCCTC**<br>**CGCCGGCGGCGTGGTTTTCGTCTTCCCAGGTCAGGGTGCTCAGTGGGAGGGCATGGCC**<br>**CGGGGCTTGCTCTCGGTCCCCGTCTTCGCCGAGTCGATCGCCGAGTGCGATGCGGTGT**<br>**TGTCGGAGGTGGCCGGGTTCTCGGCCTCCGAAGTGCTGGAGCAGCGTCCGGACGCGCC**<br>**GTCGCTGGAGCGGGTCGACGTCGTACAGCCGGTGTTGTTCTCCGTGATGGTGTCGCTG**<br>**GCGCGGCTGTGGGGCGCTTGCGGAGTCAGCCCCTCGGCCGTCATCGGCCATTCGCAGG**<br>**GCGAGATCGCCGCCGCGGTGGTGGCCGGGGTGTTGTCGCTGGAGGACGGCGTGCGCGT**<br>**CGTGGCCCTGCGCGCGAAGGCGTTGCGTGCGCTGGCGGGCAAGGGCGGCATGGTCTCG**<br>**TTGGCGGCTCCCGGTGAACGCGCCCGCGCGCTGATCGCACCGTGGGAGGACCGGATCT**<br>**CCGTCGCGGCGGTCAACTCCCCGTCCTCGGTCGTGGTCTCCGGCGATCCGGAGGCGCT**<br>**GGCCGAACTCGTCGCACGTTGCGAGGACGAGGGCGTGCGCGCCAAGACGCTCCCGGTG**<br>**GACTACGCCTCGCACTCCCGCCACGTCGAGGAGATCCGCGAGACGATCCTCGCCGACC**<br>**TCGACGGCATCTCCGCGCGGCGTGCCGCCATCCCGCTCTACTCCACGCTGCACGGCGA**<br>**ACGGCGCGACGGCGCCGACATGGGTCCGCGGTACTGGTACGACAACCTGCGCTCCCAG**<br>**GTGCGCTTCGACGAGGCGGTCTCGGCCGCCGTCGCCGACGGTCACGCCACCTTCGTCG**<br>**AGATGAGCCCGCACCCGGTGCTCACCGCGGCGGTGCAGGAGATCGCCGCGGACGCCGT**<br>**GGCCATCGGGTCGCTGCACCGCGACACCGCGGAGGAGCACCTGATCGCCGAGCTCGCC**<br>**CGGGCGCACGTGCACGGCGTGGCCGTGGACTGGCGGAACGTCTTCCCGGCGGCACCTC**<br>**CGGTGGCG**CTGCCCAACTACCCGTTCGAGCCCCAGCGGTACTGGCTCGCGCCGGAGGT<br>GTCCGACCAGCTCGCCGACAGCCGCTACCGCGTCGACTGGCGACCGCTGGCCACCACG<br>CCGGTGGACCTGGAAGGCGGCTTCCTGGTCCACGGGTCCGCACCGGAGTCGCTGACCA<br>GCGCAGTCGAGAAGGCCGGAGGCCGCGTCGTGCCGGTCGCCTCGGCCGACCGCGAAGC<br>GCTCGCGGCGGCCCTGCGGGAGGTGCCGGGCGAGGTCGCCGGCGTGCTCTCGGTCCAC<br>ACCGGCGCCGCAACGCACCTCGCCCTGCACCAGTCGCTGGGTGAGGCCGGCGTGCGGG<br>CCCCGCTCTGGCTGGTCACCAGCCGAGCGGTCGCGCTCGGGGAGTCCGAGCCGGTCGA<br>TCCCGAGCAGGCGATGGTGTGGGGTCTCGGGCGCGTCATGGCCTGGAGACCCCGGAA<br>CGGTGGGGCGGTCTGGTGGACCTGCCCGCCGAACCCGCGCCGGGGGACGGCGAGGCGT

	TCGTCGCCTGCCTCGGCGCGGACGGCCACGAGGACCAGGTCGCGATCCGTGACCACGC<br>CCGCTACGGCCGCCGCCTCGTCCGCGCCCCGCTGGGCACCCGCGAGTCGAGCTGGGAG<br>CCGGCGGGCACGGCGCTGGTCACCGGCGGCACCGGTGCGCTCGGCGGCCACGTCGCCC<br>GCCACCTCGCCAGGTGCGGGGTGGAGGACCTGGTGCTGGTCAGCAGGCGCGGCGTCGA<br>CGCTCCCGGCGCGGCCGAGCTGGAAGCCGAACTGGTCGCCCTCGGCGCGAAGACGACC<br>ATCACCGCCTGCGACGTGGCCGACCGCGAGCAGCTCTCCAAGCTGCTGGAAGAACTGC<br>GCGGGCAGGGACGTCCGGTGCGGACCGTCGTGCACACCGCCGGGGTGCCCGAATCGAG<br>GCCGCTGCACGAGATCGGCGAGCTGGAGTCGGTCTGCGCGGCGAAGGTGACCGGGGCC<br>CGGCTGCTCGACGAGCTGTGCCCGGACGCCGAGACCTTCGTCCTGTTCTCGTCCGGAG<br>CGGGGGTGTGGGGCAGTGCGAACCTCGGCGCCTACTCCGCGGCCAACGCCTACCTCGA<br>CGCGCTGGCCCACCGCCGCCGTGCGGAAGGCCGTGCGGCGACGTCCGTCGCGTGGGGC<br>GCCTGGGCGGGCGAGGGCATGGCCACCGGCGACCTCGAGGGGCTCACCCGGCGCGGCC<br>TGCGCCCGATGGCGCCCGAGCGCGCGATCCGCGCGCTGCACCAGGCGCTGGACAACGG<br>CGACACGTGCGTTTCGATCGCCGACGTCGACTGGGAGCGCTTCGCGGTCGGCTTCACC<br>GCCGCCCGGCCGCGTCCGCTGCTGGACGAGCTCGTCACGCCGGCGGTGGGGGCCGTCC<br>CCGCGGTGCAGGCGGCCCCGGCGCGGGAGATGACGTCGCAGGAGTTGCTGGAGTTCAC<br>GCACTCGCACGTCGCGGCGATCCTCGGGCATTCCAGCCCGGACGCGGTCGGGCAGGAC<br>CAGCCGTTCACCGAGCTCGGCTTCGACTCGCTGACCGCGGTCGGGCTGCGCAACCAGC<br>TCCAGCAGGCCACCGGGCTCGCGCTGCCCGCGACCCTGGTGTTCGAGCACCCCACGGT<br>CCGCAGGTTGGCCGACCACATAGGACAGCAGCTCGACAGCGGGACTCCCGCCCGGGAA<br>GCGAGCAGCGCTCTTCGCGACGGCTACCGGCAGGCGGGCGTGTCGGGCAGGGTCCGGT<br>CCTACCTCGACCTGCTGGCGGGGCTGTCGGACTTCCGCGAGCACTTCGACGGCTCCGA<br>CGGGTTCTCCCTCGATCTCGTGGACATGGCCGACGGTCCCGGAGAGGTCACGGTGATC<br>TGCTGCGCGGGAACGGCGGCGATCTCCGGTCCGCACGAGTTCACCCGGCTCGCCGGGG<br>CGCTGCGCGGAATCGCTCCGGTTCGGGCCGTGCCCCAGCCCGGCTACGAGGAGGGCGA<br>ACCTCTGCCGTCGTCGATGGCGGCGGTGGCGGCGGTGCAGGCCGATGCGGTCATCAGG<br>ACACAGGGGGACAAGCCGTTCGTGGTGGCCGGTCACTCCGCGGGGGCACTGATGGCCT<br>ACGCGCTGGCGACCGAACTGCTCGATCGCGGGCACCCGCCACGCGGTGTCGTCCTGAT<br>CGACGTCTACCCGCCCGGTCACCAGGACGCGATGAACGCCTGGCTGGAGGAGCTGACC<br>GCCACGCTGTTCGACCGCGAGACGGTGCGGATGGACGACACCAGGCTCACCGCCCTGG<br>GCGCCTACGACCGCCTCACCGGTCAGTGGCGACCCCGGGAAACCGGGCTGCCGACGCT<br>GCTGGTCAGCGCCGGCGAGCCGATGGGTCCGTGGCCCGACGACAGCTGGAAGCCGACG<br>TGGCCCTTCGAGCACGACACCGTCGCCGTCCCCGGCGACCACTTCACGATGGTGCAGG<br>AACACGCCGACGCGATCGCGCGGCACATCGACGCCTGGCTGGGCGGAGGGAATTCAAG<br>A

Supplemental Table 8. Primers for DEBS PKS construction and mutagenesis. GA = Gibson Assembly. RTH = 'Round-the-horn mutagenesis.[1] RTH primers have 5' phosphates.

Primer Name	5'→3' Primer Sequence	Primer Function
AT_Motif.Gib1	CACTCCCGCCACGTCGAG	Amplifies Ery6TE without AT motifs for GA
AT_Motif.Gib2	CTCCAGCGACGGCGCG	
AT_Motif.FOR	ACGCGCCGTCGCTGG	Amplifies motif fragments for GA
AT_Motif.REV	TCGACGTGGCGGGAGTG	
Ery6V742A.FOR	GCGGACTACGCCTCGCACTCCC	Ery6 V742A RTH
Ery6V742A.REV	CGGGAGCGTCTTGGCGC	
Ery6Y744RGV.RTH1	SKGGCCTCGCACTCCCGC	Ery6TE Y744R/G RTH
Ery6Y744RGV.RTH2	GTCCACCGGGAGCGTCTTG	
Ery6L676V.FOR	GTGGCGGGCAAGGGCG	Ery6 L676V RTH
Ery6L676V.REV	CGCACGCAACGCCTTCG	
Ery6L673G.FOR	GGACGTGCGCTGGCGGG	Ery6 L673G RTH
Ery6L673G.REV	CGCCTTCGCGCGCAG	
Ery6E647D.FOR	GATATCGCCGCCGCGG	Ery6 E647D RTH
Ery6E647D.REV	GCCCTGCGAATGGCCGAT	
Ery6L797X.FOR	NNKCGCTCCCAGGTGCGC	Ery6 L797X RTH
Ery6L797X.REV	GTTGTCGTACCAGTACCGCGGA	
Ery6I648A.FOR	GCCGCCGCGGTGGTGG	Ery6 I648G/A/V RTH
Ery6I648X.REV	GNSCTCGCCCTGCGAATGGC	
Ery6H643F.FOR	TTTTCGCAGGGCGAGATCGC	Ery6 H643F RTH
Ery6H643F.REV	GCCGATGACGGCCGAGG	
Ery6H643S.FOR	TCTTCGCAGGGCGAGATCGC	Ery6 H643S RTH
Ery6H643F.REV	GCCGATGACGGCCGAGG	
Ery6L740V.FOR	GTCCCGGTGGACTACGCCTC	Ery6 L740V RTH
Ery6L740V.REV	CGTCTTGGCGCGCACG	
Ery6A707G.FOR	GGGGTCAACTCCCCGTCCTCG	Ery6 A707G RTH
Ery6A707G.REV	CGCGACGGAGATCCGGTC	
Ery6G681A.FOR	GCAATGGTCTCGTTGGCGGC	Ery6 G681A RTH
Ery6G681A.REV	GCCCTTGCCCGCCAGC	
Ery6V751A.FOR	GCCGAGGAGATCCGCGAGAC	Ery6 V751A RTH
Ery6V751A.REV	GTGGCGGGAGTGCGAGG	
Ery6S644A.FOR	GTCAGCCGTTATCGGTCATGCT	Ery6 S644A RTH
Ery6S644A.REV	CCGCAATTTCGCCCTG	
Ery6D613E.FOR	GAAGTCGTACAGCCGGTGTTG	Ery6 D613E RTH
Ery6D613E.REV	GACCCGCTCCAGCGACG	
Ery6V751A.FOR	GCCGAGGAGATCCGCGAGAC	Ery6 V751A RTH
Ery6V751A.REV	GTGGCGGGAGTGCGAGG	

Supplemental Table 9. High-resolution LC-MS parameters and gradient.

HESI Source Parameters	
Spray Voltage	3.5 kV
Capillary Temperature	350 °C
Heater Temperature	300 °C
S Lens RF Level	70 V
Sheath Gas Flow Rate	60 au
Resolution	70,000 FWHM
Scan Range	100-1000 m/z

LC Gradient

Time (min)	% B
0.0	25.0
1.0	25.0
10.0	59.0
11.0	85.0
12.0	85.0
12.5	25.0
16.5	25.0

Supplementary Figures

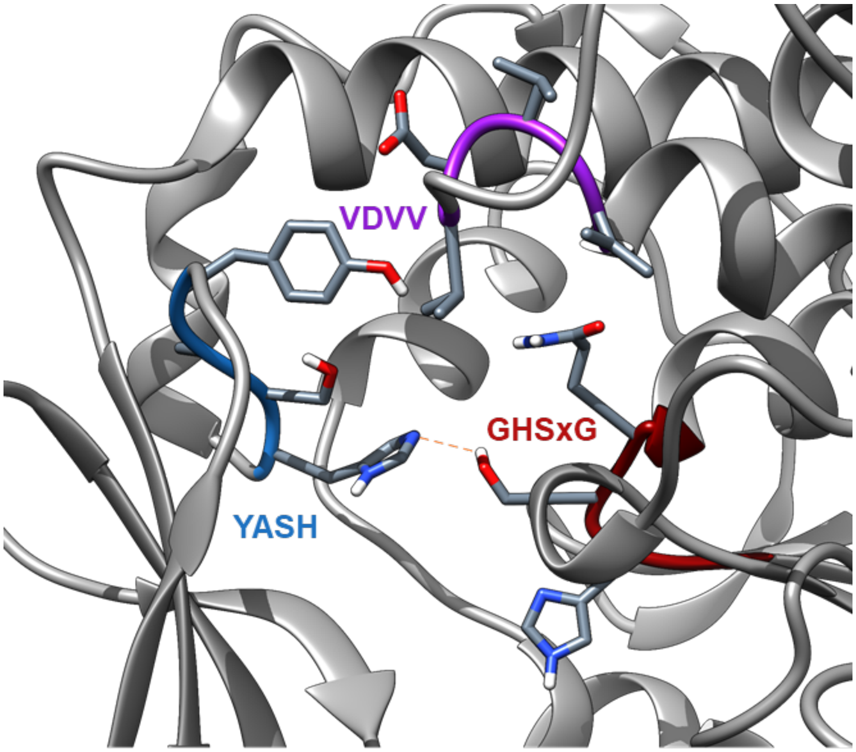

Supplemental Figure 1. Conserved motifs of PKS acyltransferases. Active site of EryAT6 with well-conserved motifs from methylmalonyl-CoA-specific ATs depicted as sticks (SeqLogos in **Supplemental Figure 9**).

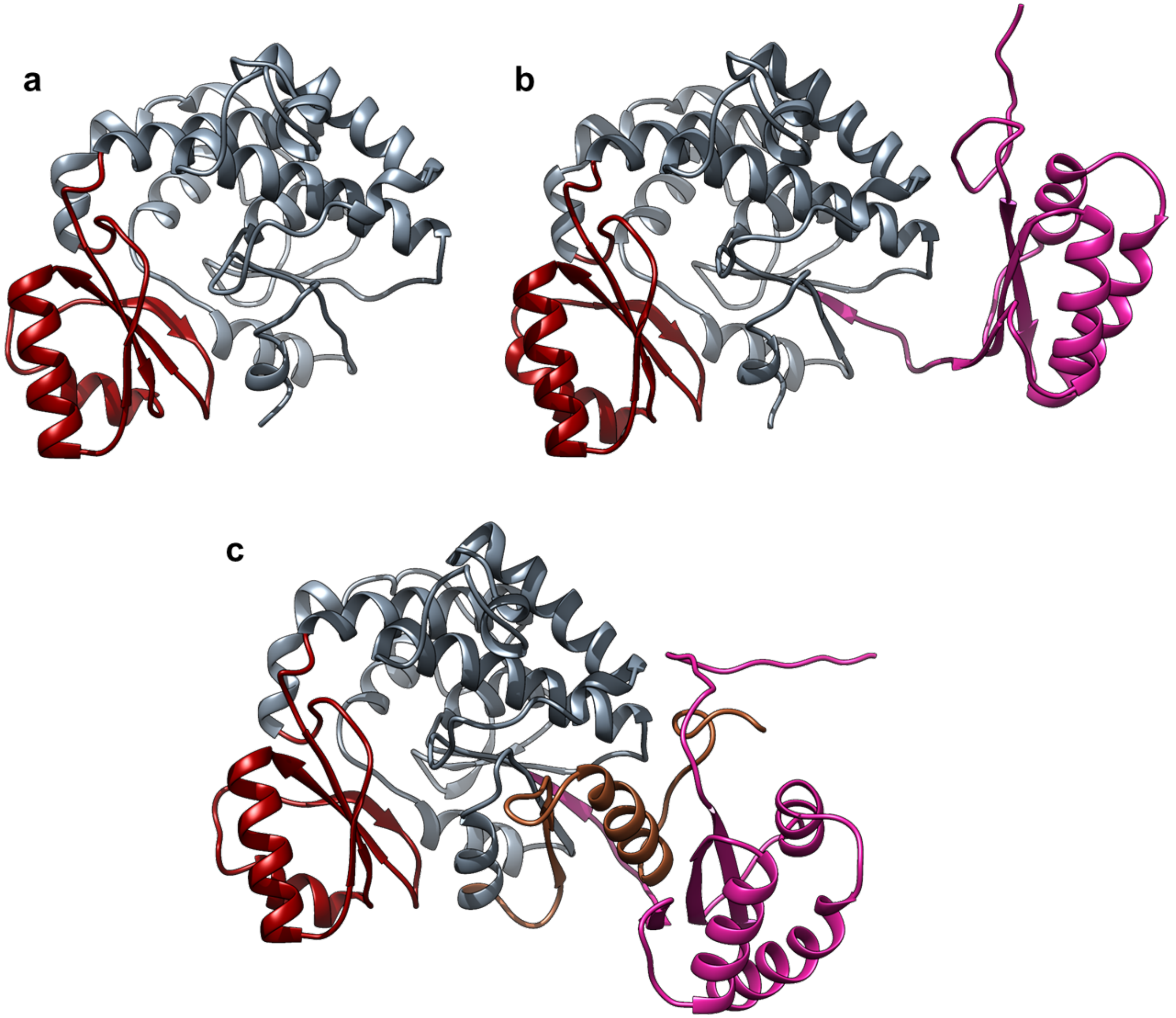

Supplemental Figure 2. Evaluation of EryAT6 models. Time-averaged wild-type EryAT6 homology model structures from MD simulations with (**a**) no linkers and a total of 278 residues, (**b**) an N-terminal linker (magenta) and a total of 387 residues, and (**c**) an N-terminal linker (magenta), a C-terminal linker (brown), and a total of 430 residues. This final model was selected for all remaining simulations, and the per-residue secondary structure over time is shown in Figure S3. The small (red) and large (grey) subunits are included in all models.

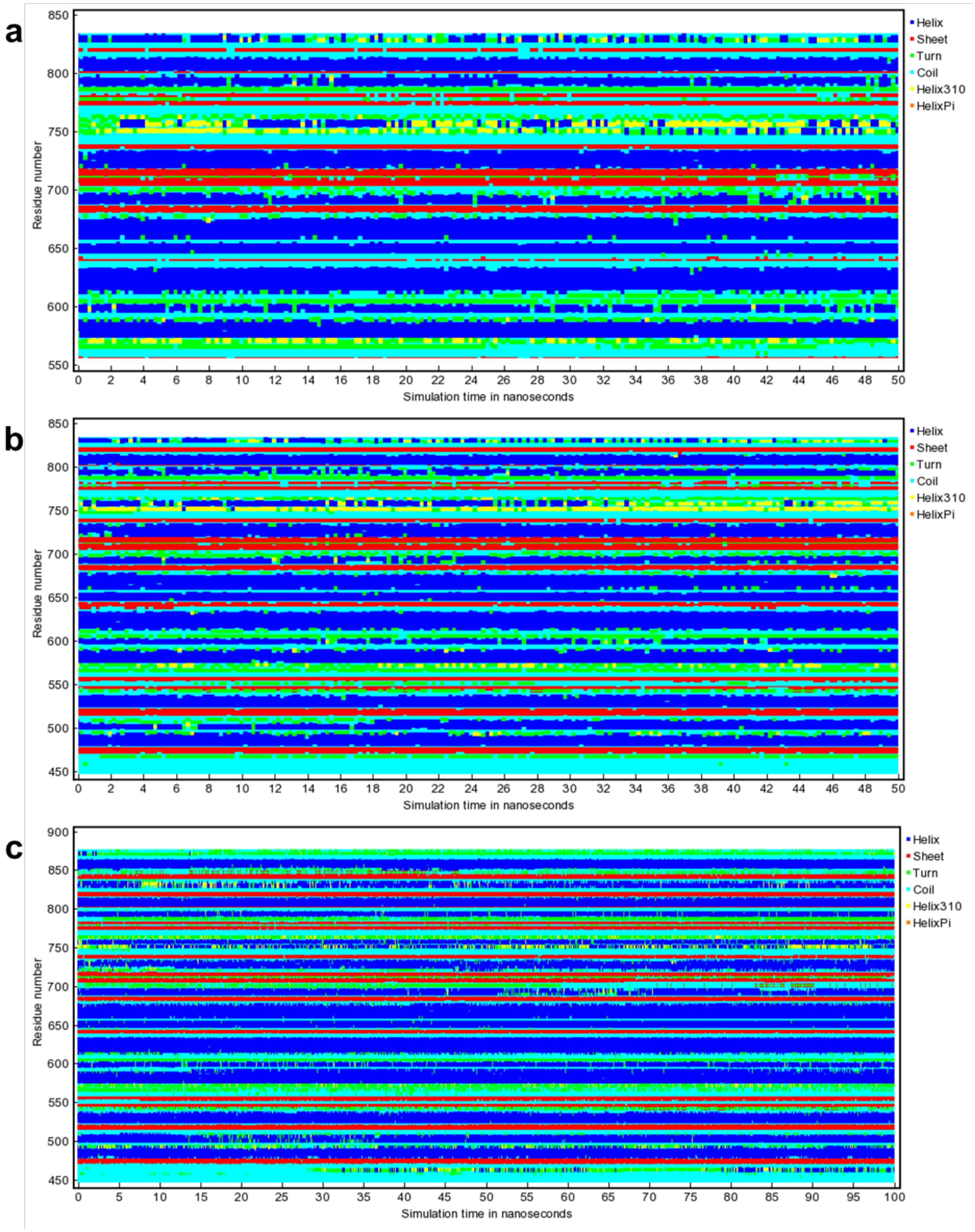

Supplemental Figure 3. Per-residue protein secondary structure of EryAT6 models. Wild-type EryAT6 homology models simulated with (**a**) no linkers and a total of 278 residues, (**b**) an N-terminal linker (magenta) and a total of 387 residues, and (**c**) an N-terminal linker (magenta), a C-terminal linker (brown), and a total of 430 residues each showed different levels of secondary structure retention over time. In the two models lacking a C-terminal linker, several secondary structures were less stable than in the two-linker model, especially in the helices near residue 750, which are important for the integrity of the active site.

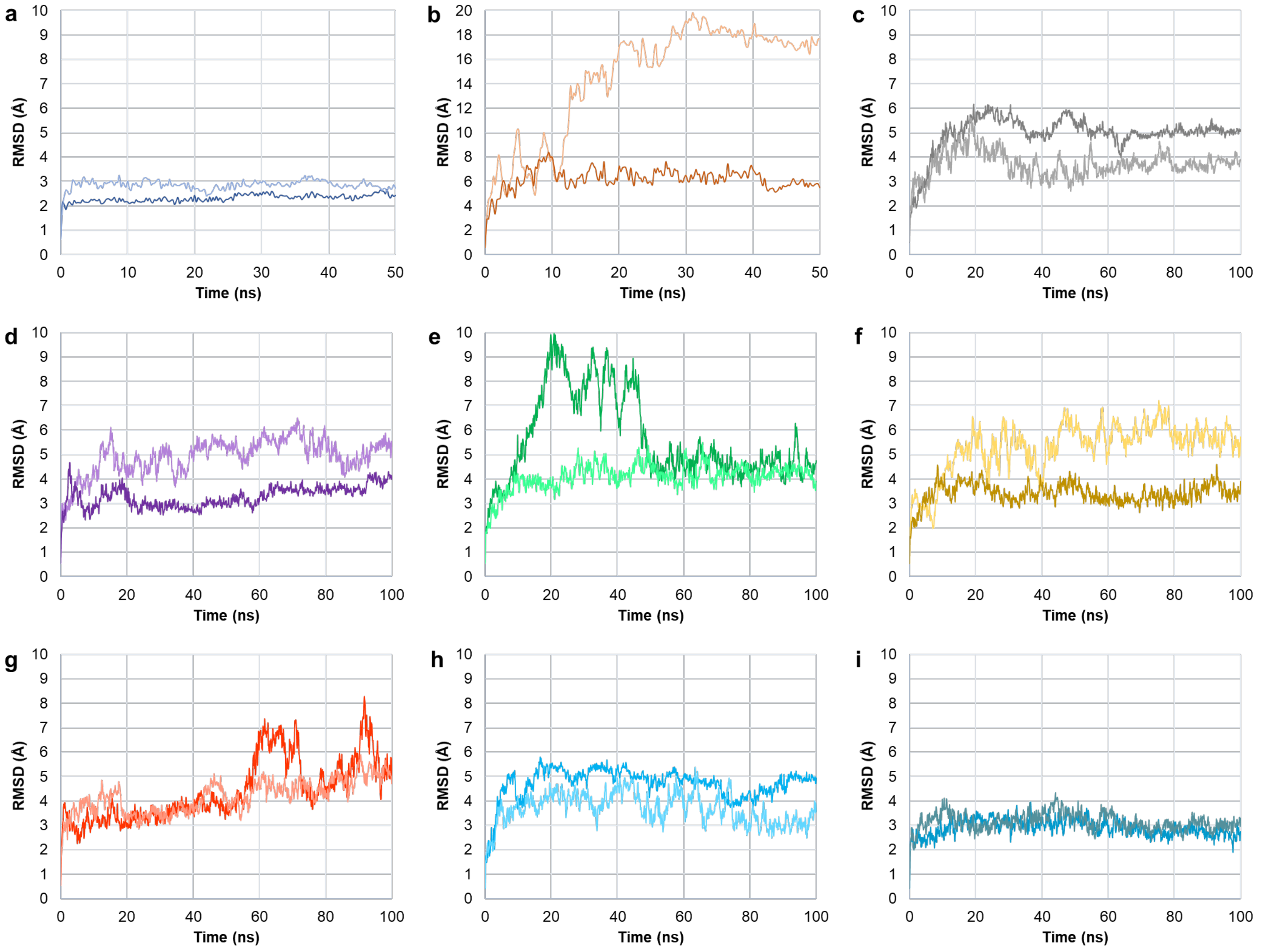

Supplemental Figure 4. RMSD of EryAT6 MD simulations. RMSD of the Cα from all residues within the AT for each of the EryAT6 models: (**a**) wild-type with no linkers. (**b**) wild-type with only the N-terminal linker. (**c**) wild-type. (**d**) V742A. (**e**) Y744R. (**f**) V742A/Y744R. (**g**) L673H. (**h**) Cin1. (**i**) Tha13. Each simulation was run in duplicate and RMSDs are shown for each simulation. Rotation of the N- and/or C-terminal linkers resulted in large RMSD shifts in some simulations.

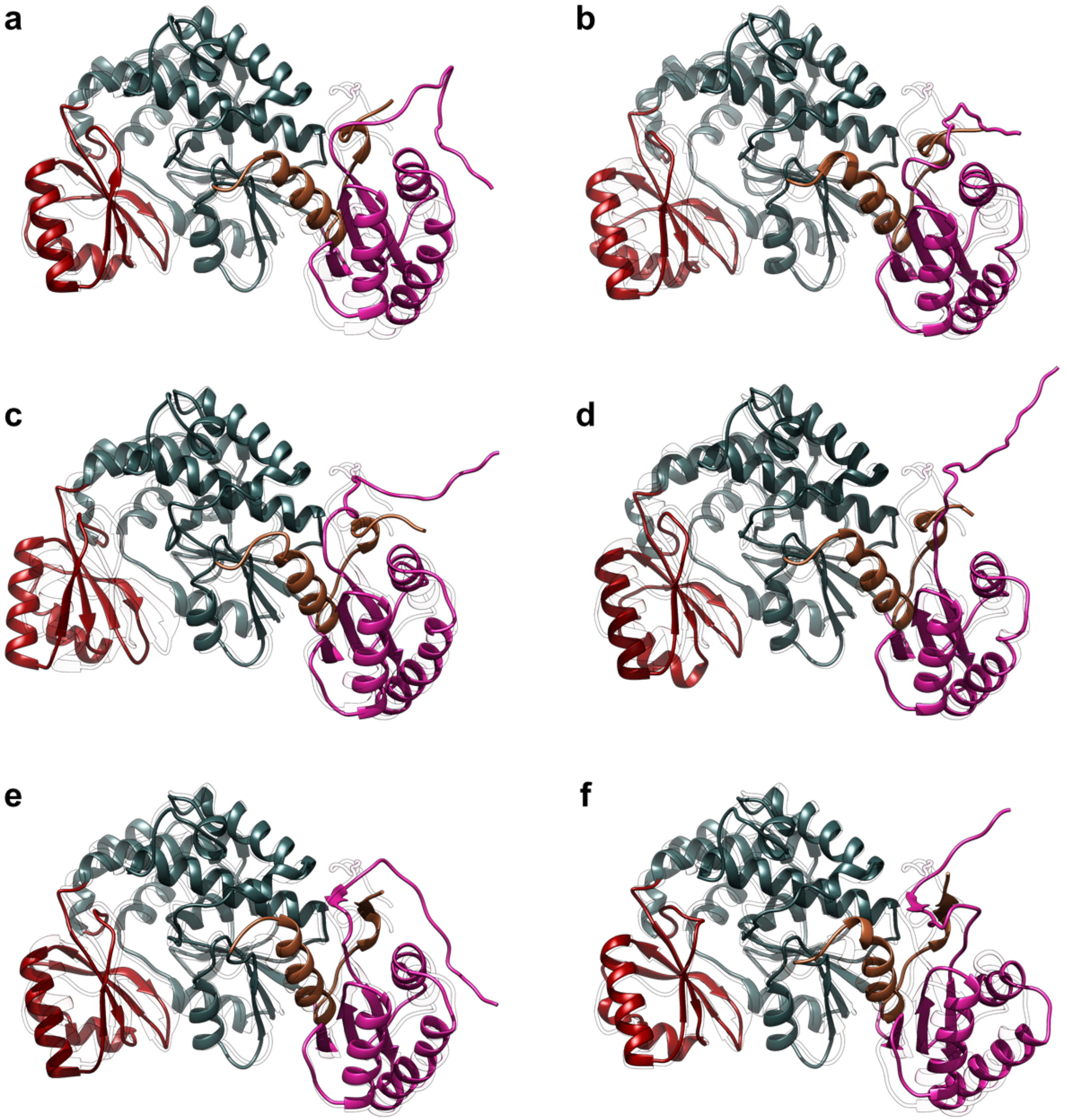

Supplemental Figure 5. Time-averaged structures of EryAT6 variants. Time-averaged structures of EryAT6 variants derived from the final 80 ns of the MD simulations are overlaid on the time-averaged wild-type EryAT6 structure (transparent). (**a**) The V742A mutant does not show a major rearrangement of the protein backbone relative to wild-type. (**b**) The Y744R mutant shows a significant increase in the width of the active site, especially in the proximity of the small subunit motif. (**c**) The double mutant V742A/Y744R shows significant perturbation of the small subunit, with little to no interaction between the catalytic residues. (**d**) The L673H mutant only shows a slight shift in the position of the small subunit relative to the large subunit. Both motif-swapped mutants, Cin1 (**e**) and Tha13 (**f**), show little difference in the average position of the two subunits, especially compared with the other two mutants containing Y744R.

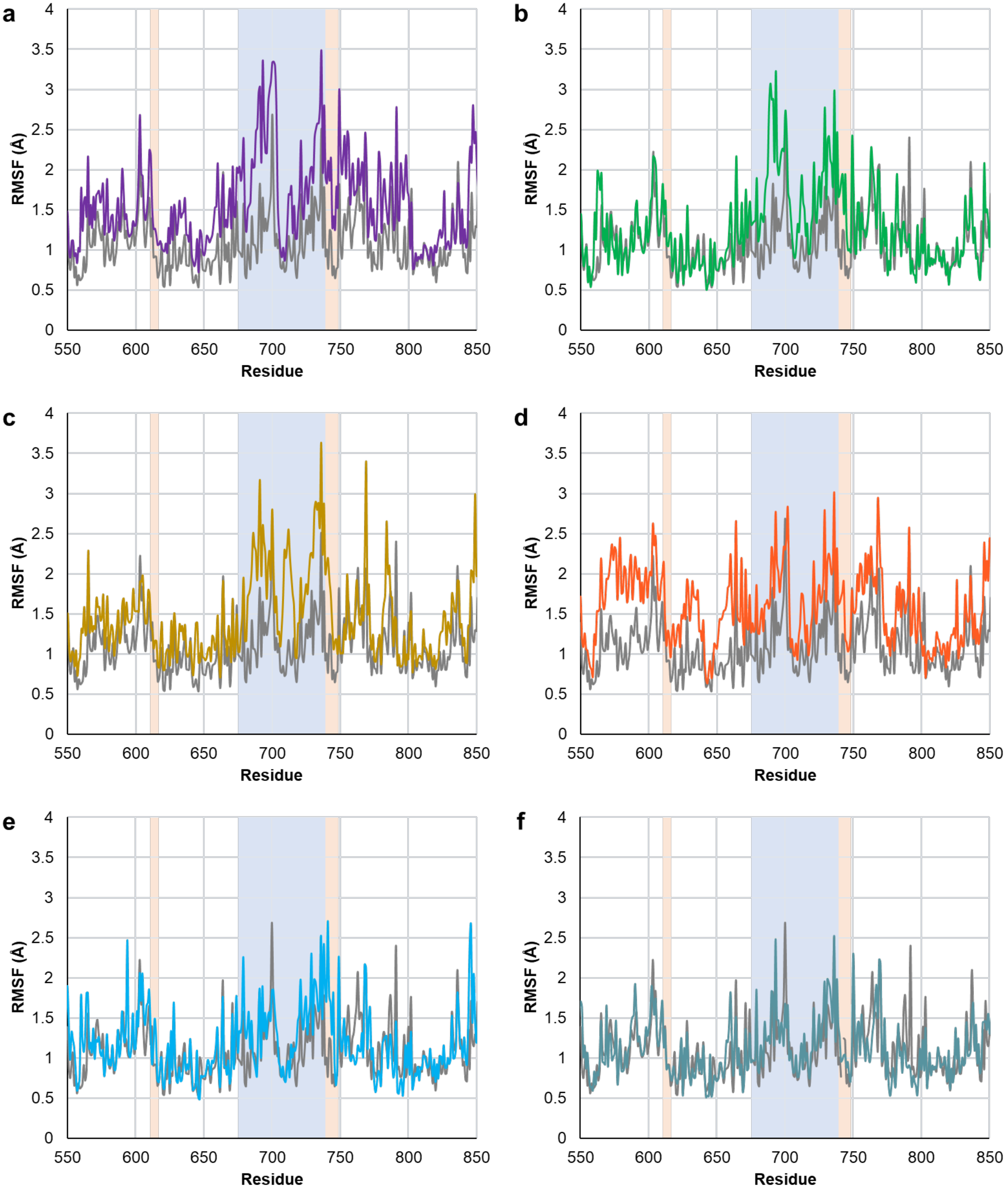

Supplemental Figure 6. RMSF plots for EryAT6 simulations. Per-residue root-mean-square fluctuation (RMSF) measurements were calculated for the final 80 ns of each 100 ns MD simulation of the EryAT6 models to allow for an equilibration period. In each panel, the grey trace represents the movement seen in the wild-type simulation, overlaid by a second trace for each mutant: (**a**) V742A, (**b**) Y744R, (**c**) V742A/Y744R, (**d**) L673H, (**e**) Cin1, and (**f**) Tha13. Higher values correspond to increased movement of a residue over the time frame. The blue box represents the residues found within the small subunit of the AT. The orange boxes highlight the residues located within the large subunit motif (LSM; 611-617) and the small subunit motif (SSM; 739-747). Linkers were excluded from the analyses for clarity.

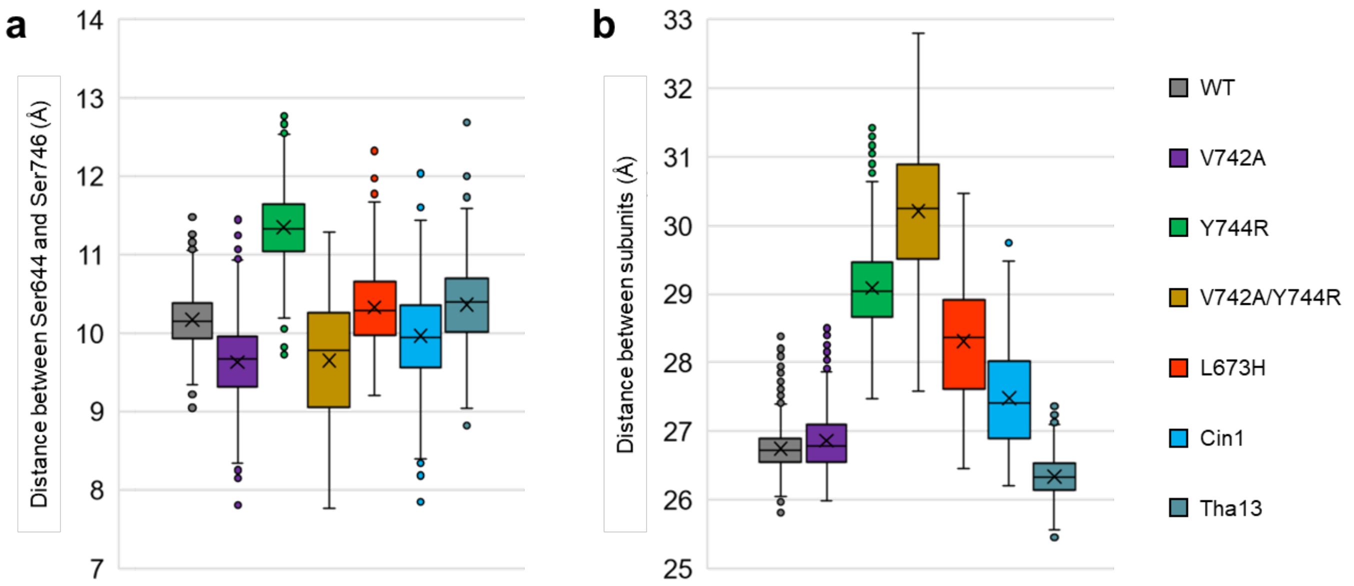

Supplemental Figure 7. Distance plots for EryAT6 simulations. Distance measurements were calculated for the final 80 ns of each 100 ns MD simulation of the EryAT6 models to allow for an equilibration period. (**a**) The distances between the catalytic Ser644 and the third residue of the YASH motif (Ser746 in wild-type) shows little variation except for a wider active site in the Y744R mutant. (**b**) The distance between the large and small subunits (by center of mass) shows a much shorter distance for the two motif-swapped mutants relative to most of the point mutants or even wild-type. The X indicates the mean. The boxes represent the range from the first to third quartiles with the center line representing the median. The whiskers represent the minima and maxima or, in the presence of outliers indicated by points, 1.5 times the interquartile range. These distances were measured every 0.1 ns over the final 80 ns (*n* = 800) of two independent simulations.

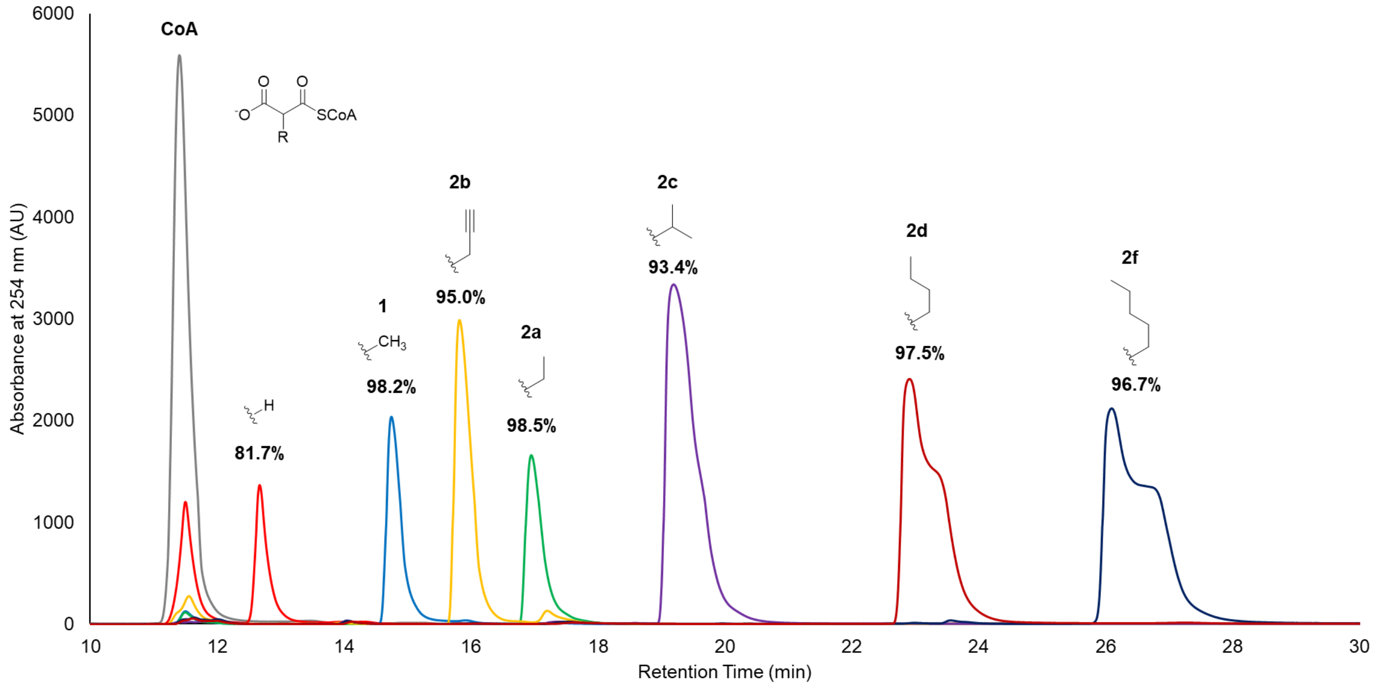

Supplemental Figure 8. Representative HPLC traces for MatB-synthesized malonyl-CoAs. Representative HPLC traces for MatB-synthesized malonyl-CoAs. Percent conversions were determined by remaining CoA peak. Individual reaction conditions are described in the methods.

a

(2S)-X-malonyl-CoA	Acyltransferase	Small Subunit Motif	Large Subunit Motif
H	Avermectin AT5	TLP-TNHAFH	QTPYAQP
Methyl	Erythromycin AT6	TLP-VDYASH	RVDVVQP
Methyl	Rifamycin AT3	RVA-VDYASH	RVDVVQP
Ethyl/Methyl	Monensin AT5	AVA-SDVAGH	RIDVVQP
Ethyl	Niddamycin AT5	PIPGVDTAGH	RVDVVQP
Allyl/Ethyl	FK506 AT4	RIA-VDCPTH	RVDVVHP
Chloroethyl	Salinosporamide AT1	AVR-VDRPGH	ATDIQQP
Butyl	Thailandin AT13	VLPGADAAGH	RLDVNQP
Hexyl	Cinnabaramide AT1	ALR-VERAGH	EGDIQQP
Benzyl	Splenocin AT1	RLR-MPAAAH	--TAAFM

b

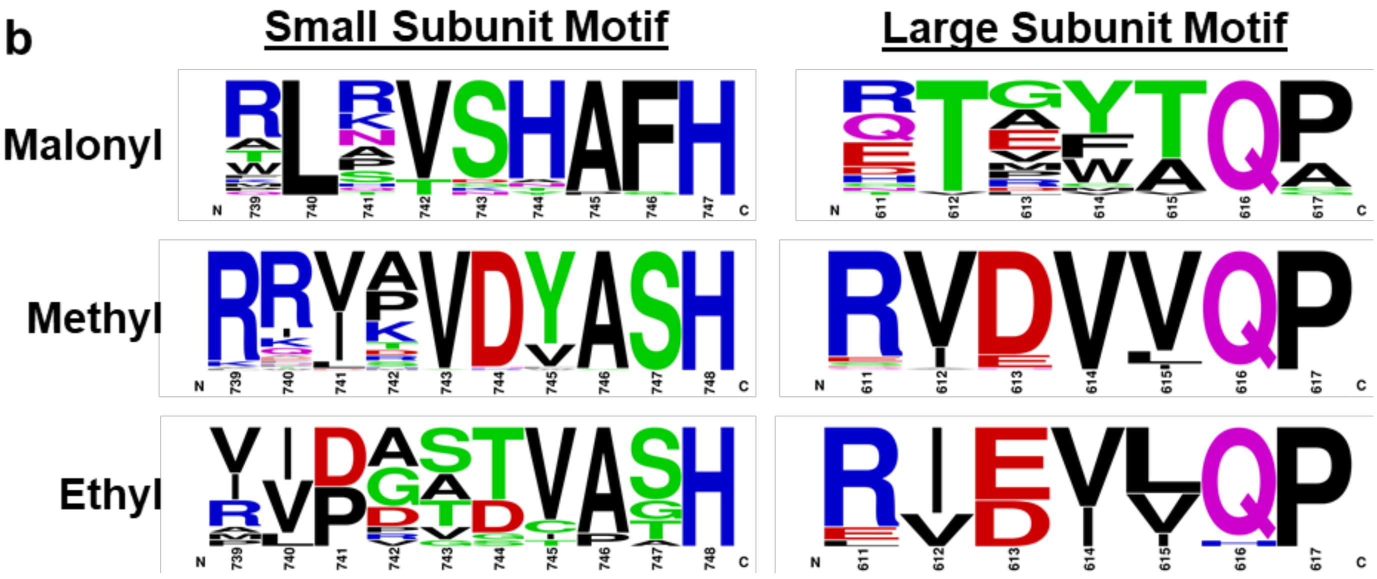

Supplemental Figure 9. Motif sequences from natural acyltransferases. (**a**) Selected examples of SSM and LSM sequences for ATs with different CoA-linked substrates. (**b**) Consensus sequences of AT domains with substrate specificities for malonyl-CoA (100 sequences), methylmalonyl-CoA (100 sequences), and ethylmalonyl-CoA (19 sequences) are shown. Sequence logos were created using WebLogo.[6]

Supplemental Figure 10. Representative mass spectra of 10-deoxymethynolide B compounds from lysate module reactions. Top panel for each compound shows the extracted ion chromatogram. Bottom panel(s) show the total ion spectra.

a

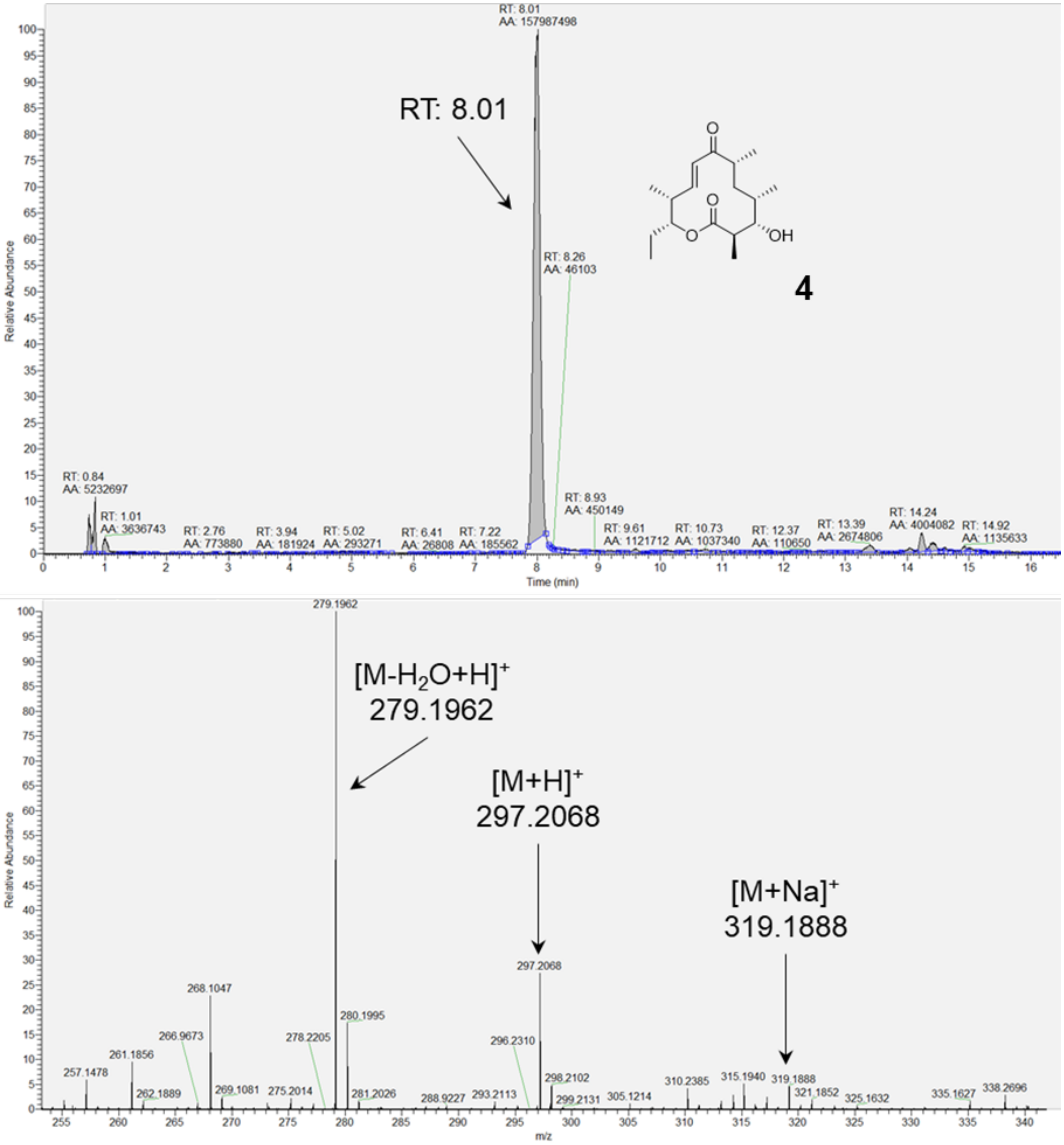

b

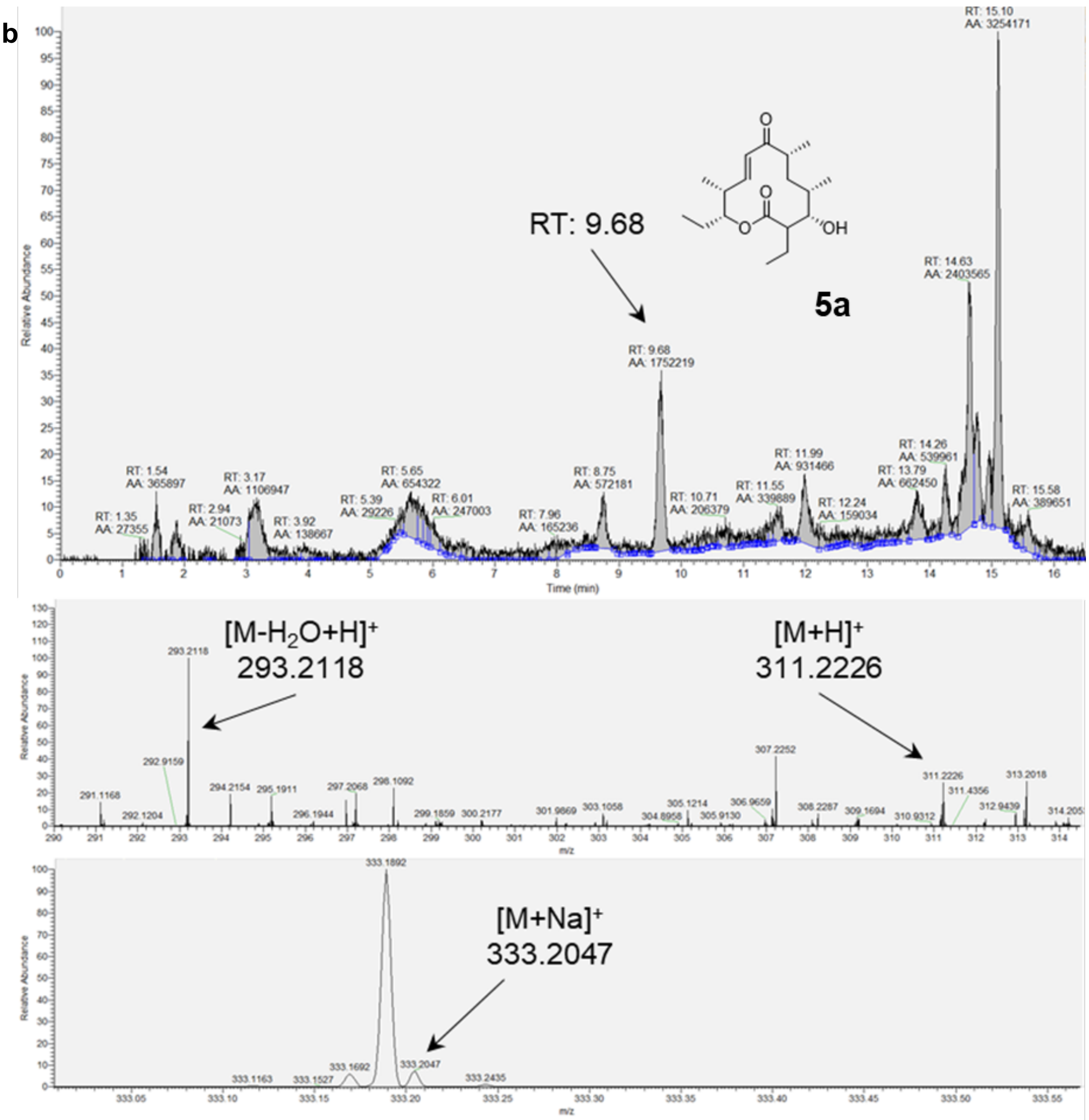
RT: 9.68
5a
[M-H2O+H]+
293.2118
[M+H]+
311.2226
[M+Na]+
333.2047
Relative Abundance
Time (min)
m/z

c

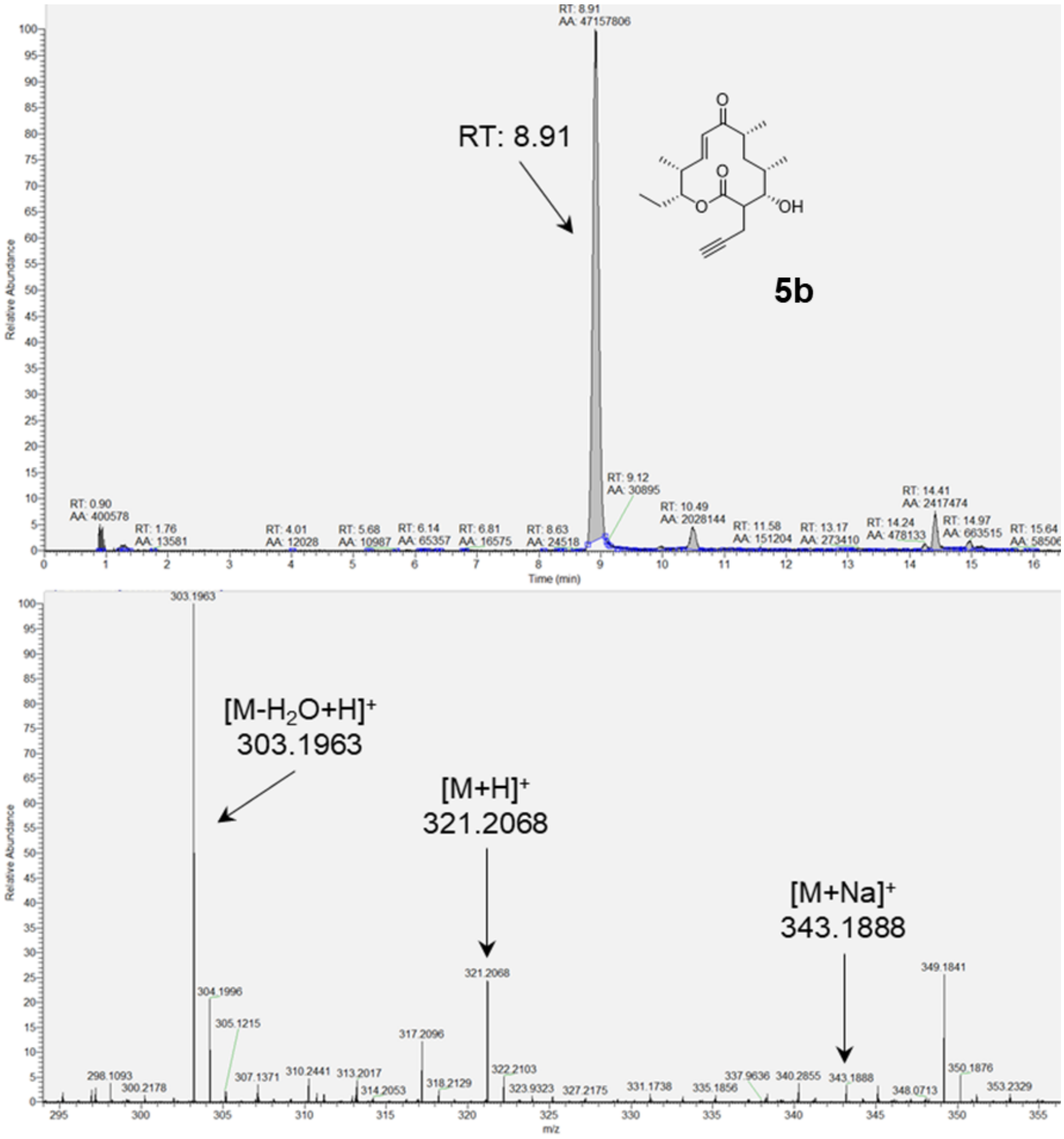
RT: 8.91
5b
[M-H$_2$O+H]$^+$
303.1963
[M+H]$^+$
321.2068
[M+Na]$^+$
343.1888
Relative Abundance
Time (min)
m/z

d

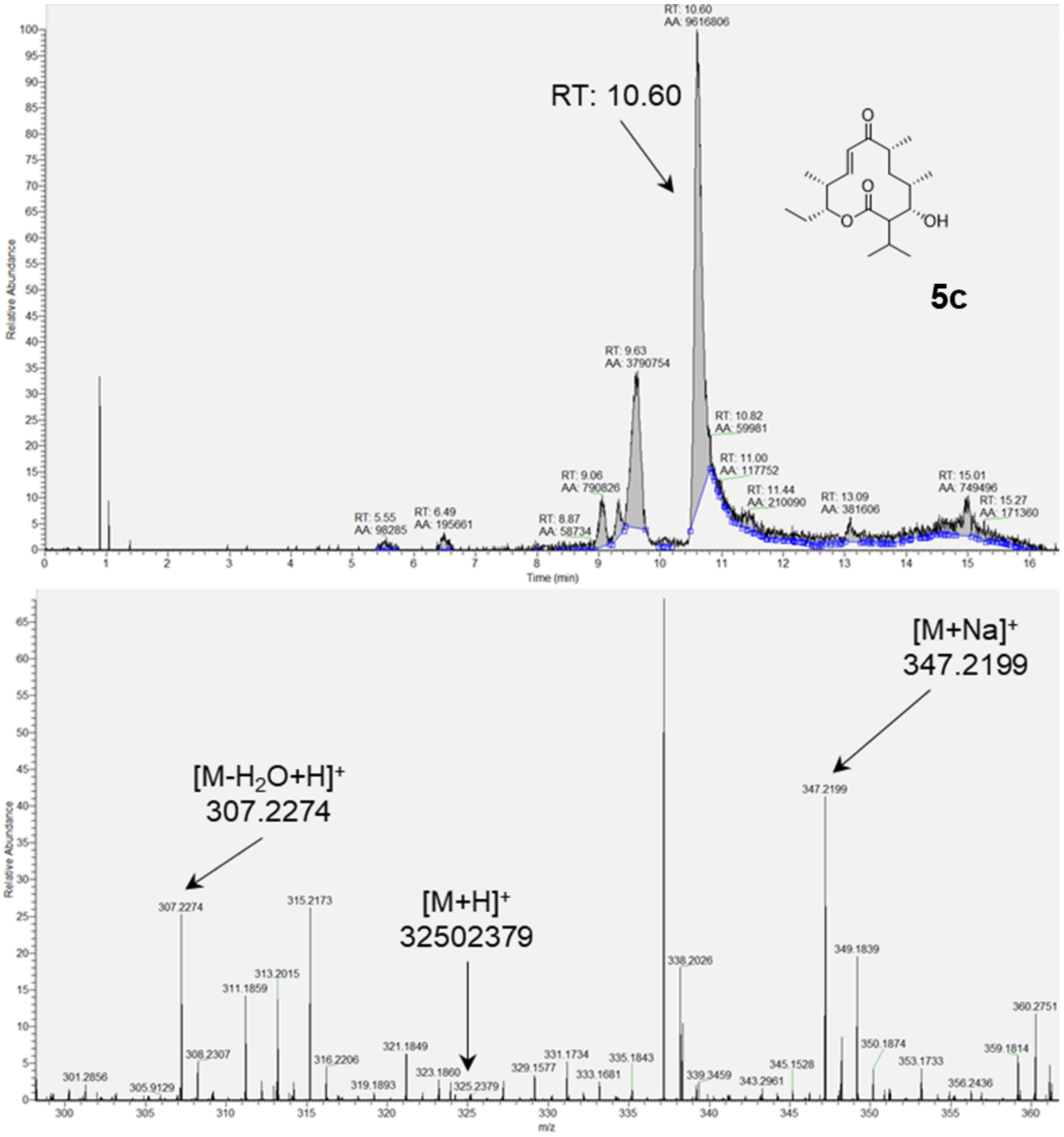
RT: 10.60
5c
[M-H2O+H]+
307.2274
[M+H]+
32502379
[M+Na]+
347.2199
Relative Abundance
Time (min)
m/z

e

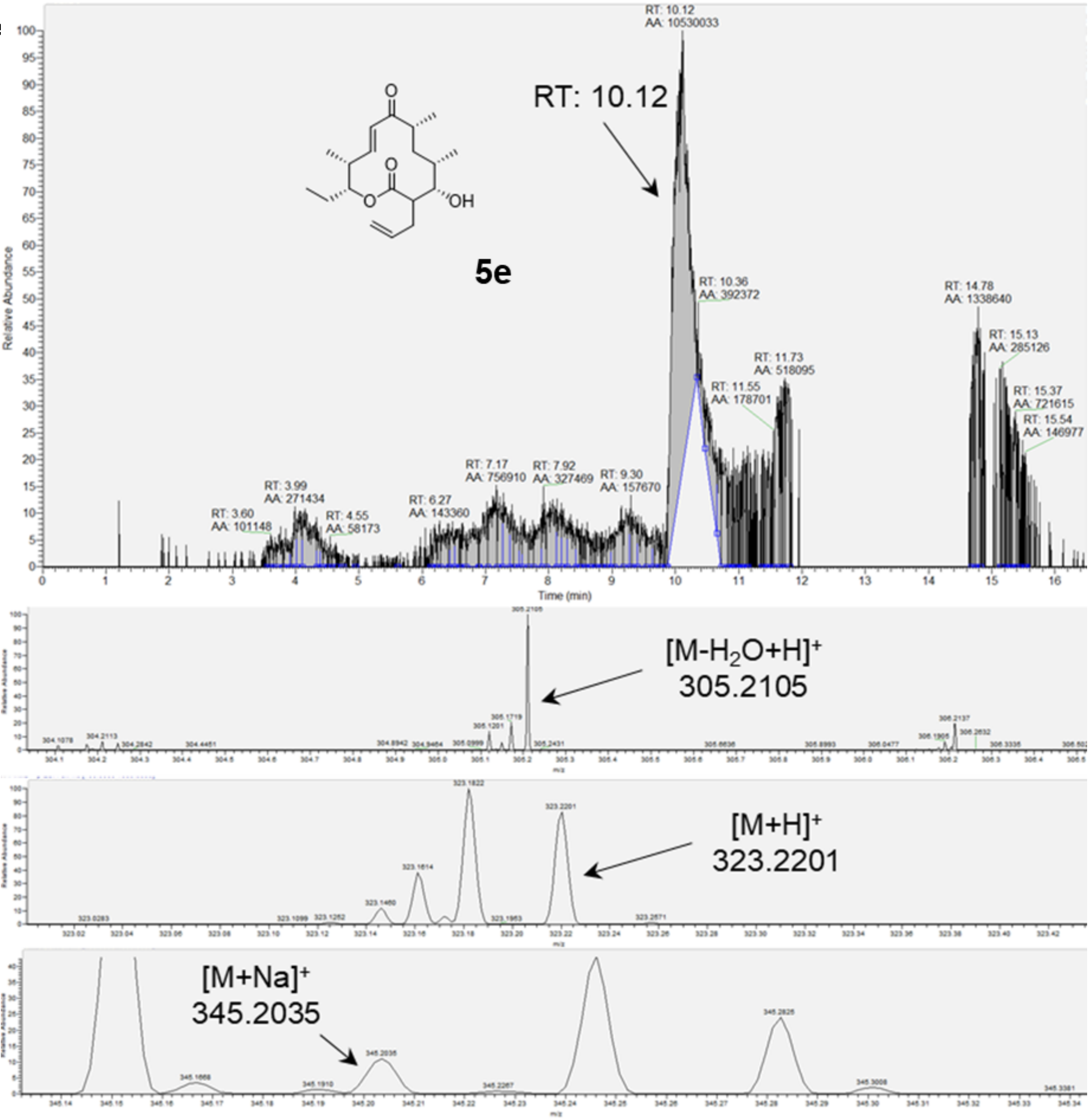

RT: 10.12
AA: 10530033
RT: 10.12
5e
OH
O
RT: 10.36
AA: 392372
RT: 11.73
AA: 518095
RT: 11.55
AA: 178701
RT: 14.78
AA: 1338640
RT: 15.13
AA: 285126
RT: 15.37
AA: 721615
RT: 15.54
AA: 146977
RT: 3.60
AA: 101148
RT: 3.99
AA: 271434
RT: 4.55
AA: 58173
RT: 6.27
AA: 143360
RT: 7.17
AA: 756910
RT: 7.92
AA: 327469
RT: 9.30
AA: 157670
Relative Abundance
Time (min)
[M-H2O+H]+
305.2105
[M+H]+
323.2201
[M+Na]+
345.2035

Supplemental Figure 11. Representative mass spectra of keto-10-deoxymethynolide B compounds from lysate module reactions. Top panel for each compound shows the extracted ion chromatogram. Bottom panel(s) show the total ion spectra.

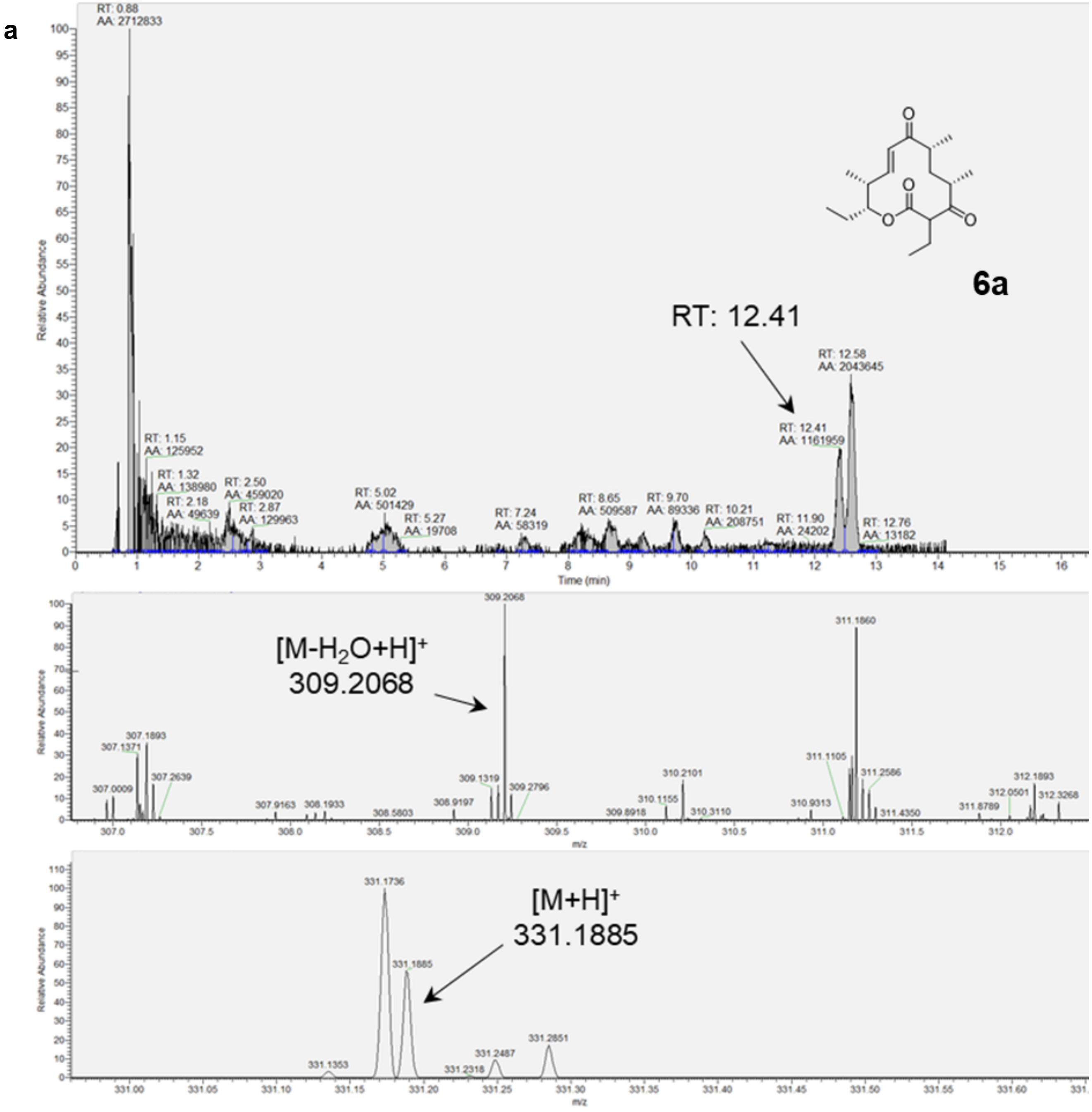

b

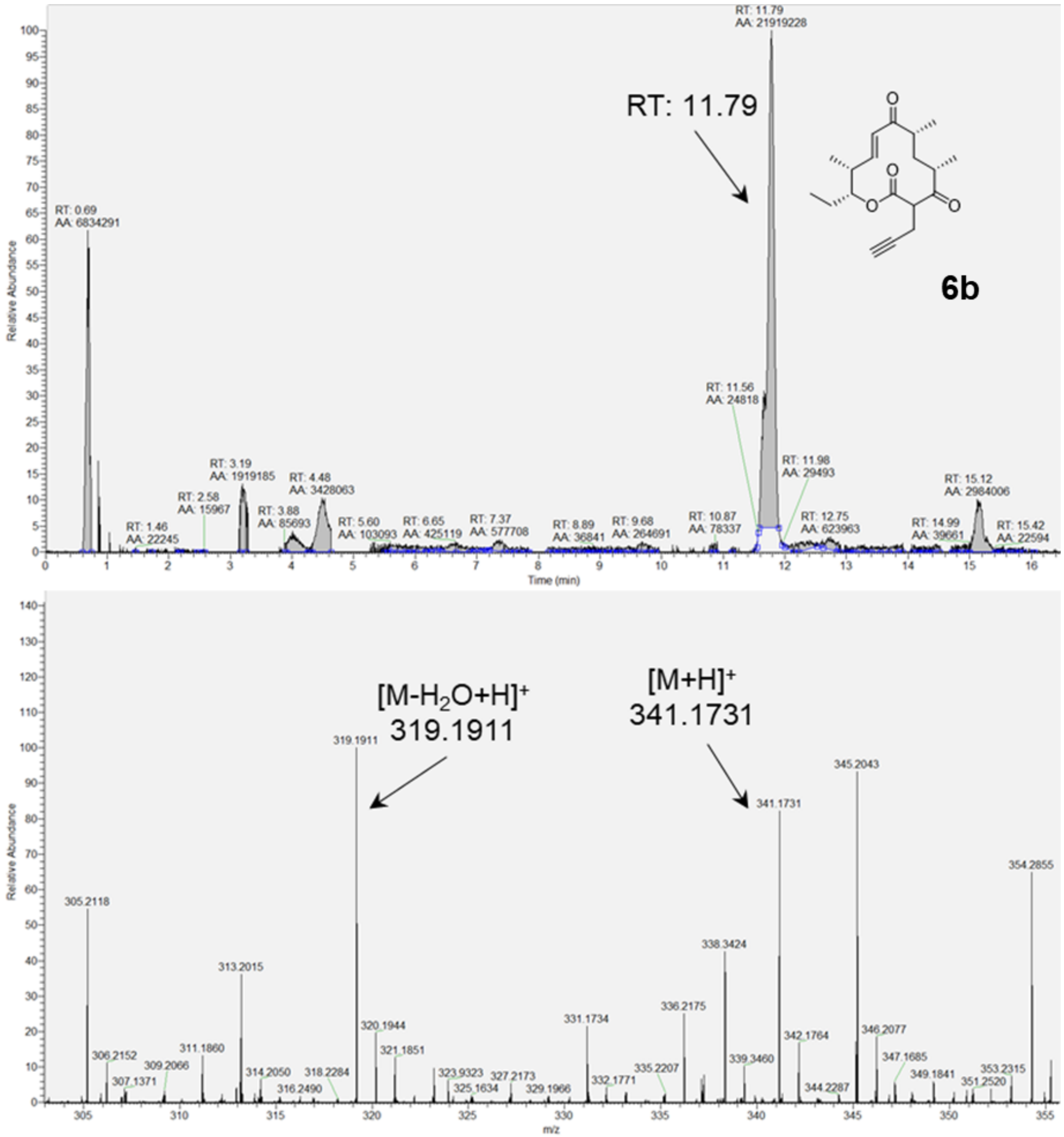
RT: 11.79
AA: 21919228
RT: 11.79
6b
RT: 0.69
AA: 6834291
RT: 1.46
AA: 22245
RT: 2.58
AA: 15967
RT: 3.19
AA: 1919185
RT: 3.88
AA: 85693
RT: 4.48
AA: 3428063
RT: 5.60
AA: 103093
RT: 6.65
AA: 425119
RT: 7.37
AA: 577708
RT: 8.89
AA: 36841
RT: 9.68
AA: 264691
RT: 10.87
AA: 78337
RT: 11.56
AA: 24818
RT: 11.98
AA: 29493
RT: 12.75
AA: 623963
RT: 14.99
AA: 39661
RT: 15.12
AA: 2984006
RT: 15.42
AA: 22594
Relative Abundance
Time (min)
[M-H2O+H]+
319.1911
[M+H]+
341.1731
305.2118
306.2152
307.1371
309.2066
311.1860
313.2015
314.2050
316.2490
318.2284
319.1911
320.1944
321.1851
323.9323
325.1634
327.2173
329.1966
331.1734
332.1771
335.2207
336.2175
338.3424
339.3460
341.1731
342.1764
344.2287
345.2043
346.2077
347.1685
349.1841
351.2520
353.2315
354.2855
Relative Abundance
m/z

c

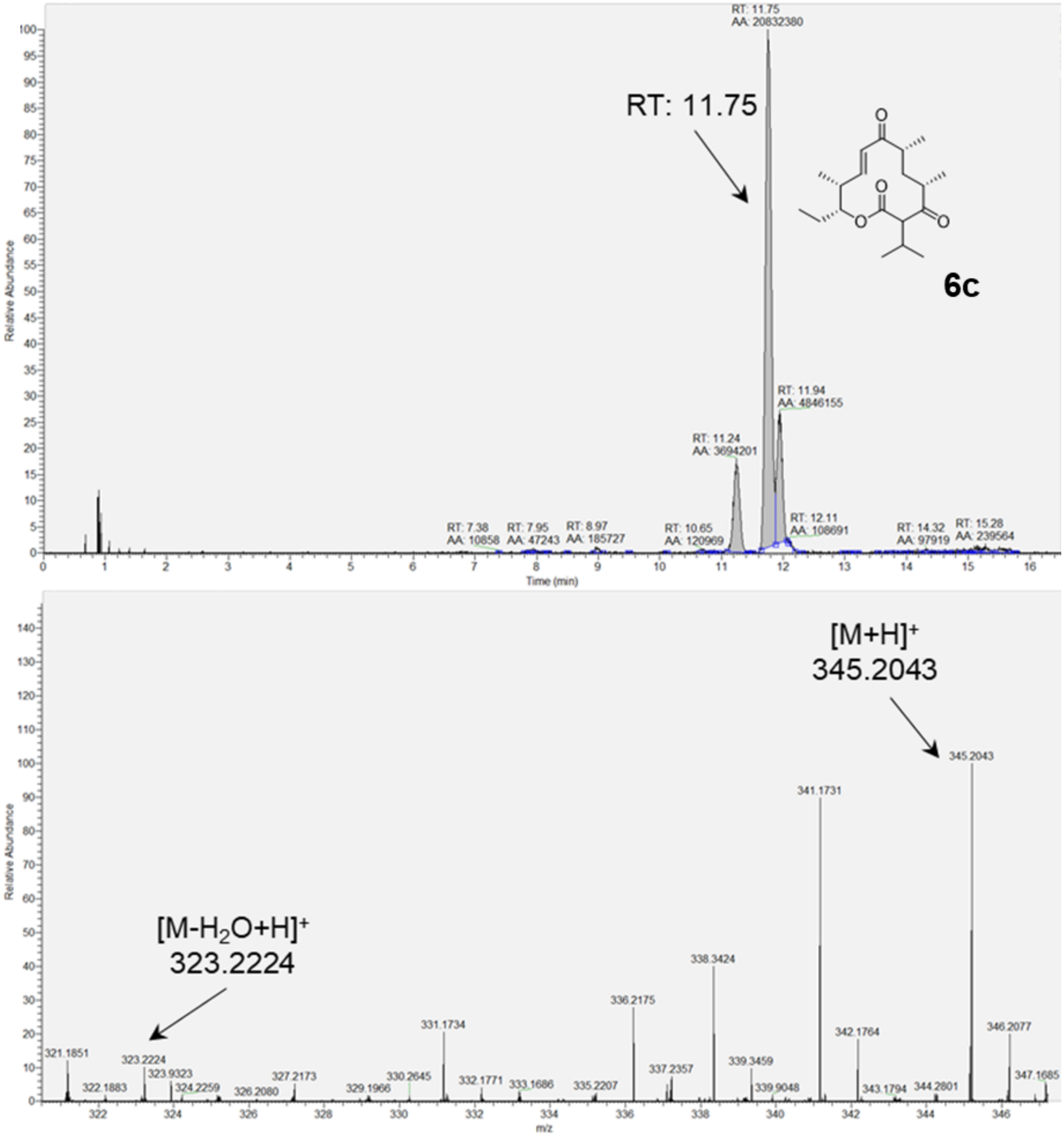

RT: 11.75
AA: 20832380
RT: 11.75
6c
RT: 11.94
AA: 4846155
RT: 11.24
AA: 3694201
RT: 12.11
AA: 108691
RT: 7.38
AA: 10858
RT: 7.95
AA: 47243
RT: 8.97
AA: 185727
RT: 10.65
AA: 120969
RT: 14.32
AA: 97919
RT: 15.28
AA: 239564
Relative Abundance
Time (min)
[M+H]+
345.2043
[M-H2O+H]+
323.2224
345.2043
341.1731
338.3424
336.2175
331.1734
342.1764
346.2077
321.1851
323.2224
323.9323
324.2259
322.1883
326.2080
327.2173
329.1966
330.2645
332.1771
333.1686
335.2207
337.2357
339.3459
339.9048
343.1794
344.2801
347.1685
Relative Abundance
m/z

d

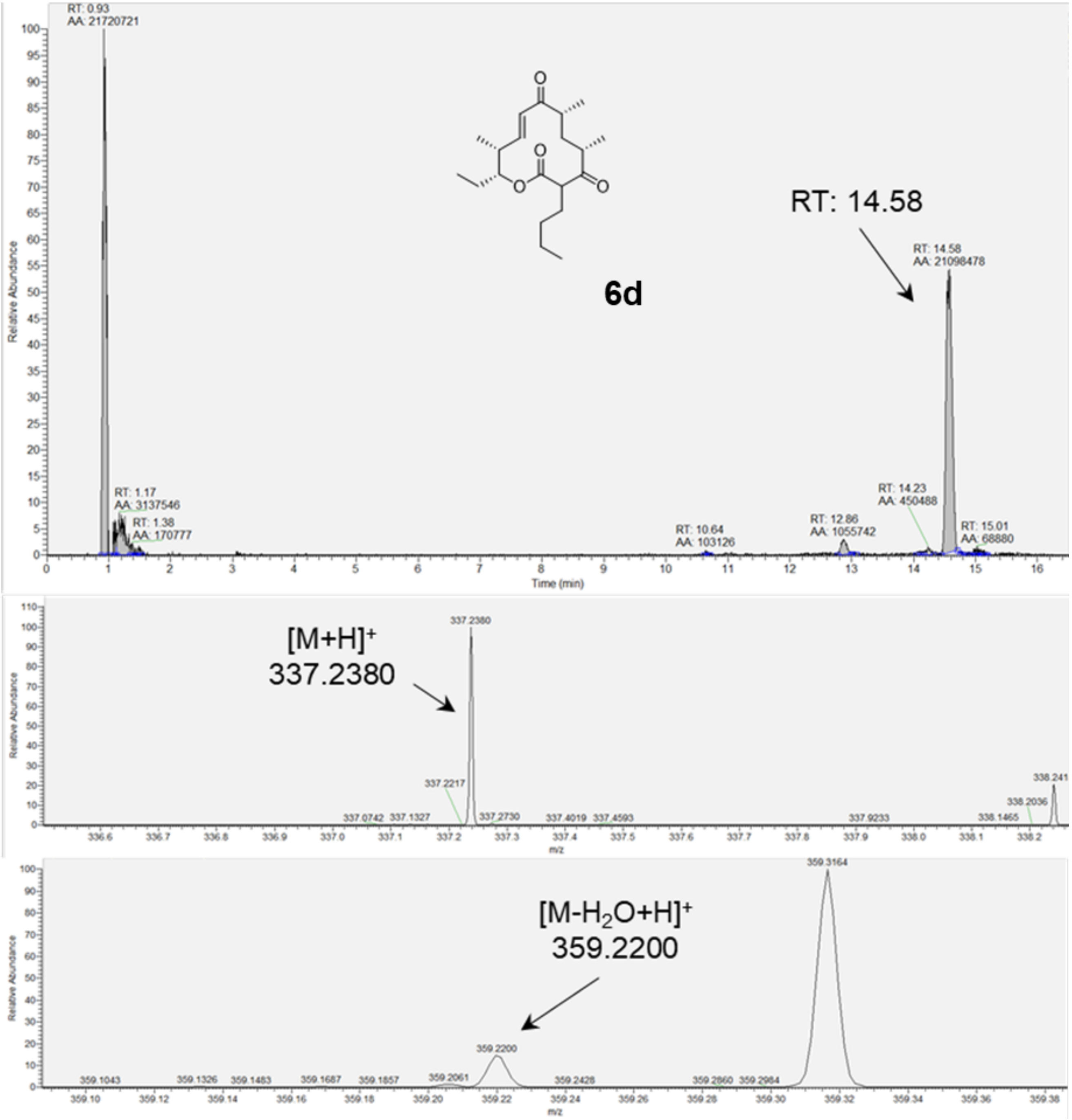

6d
RT: 14.58
[M+H]+
337.2380
[M-H2O+H]+
359.2200
Relative Abundance
Time (min)
m/z

e

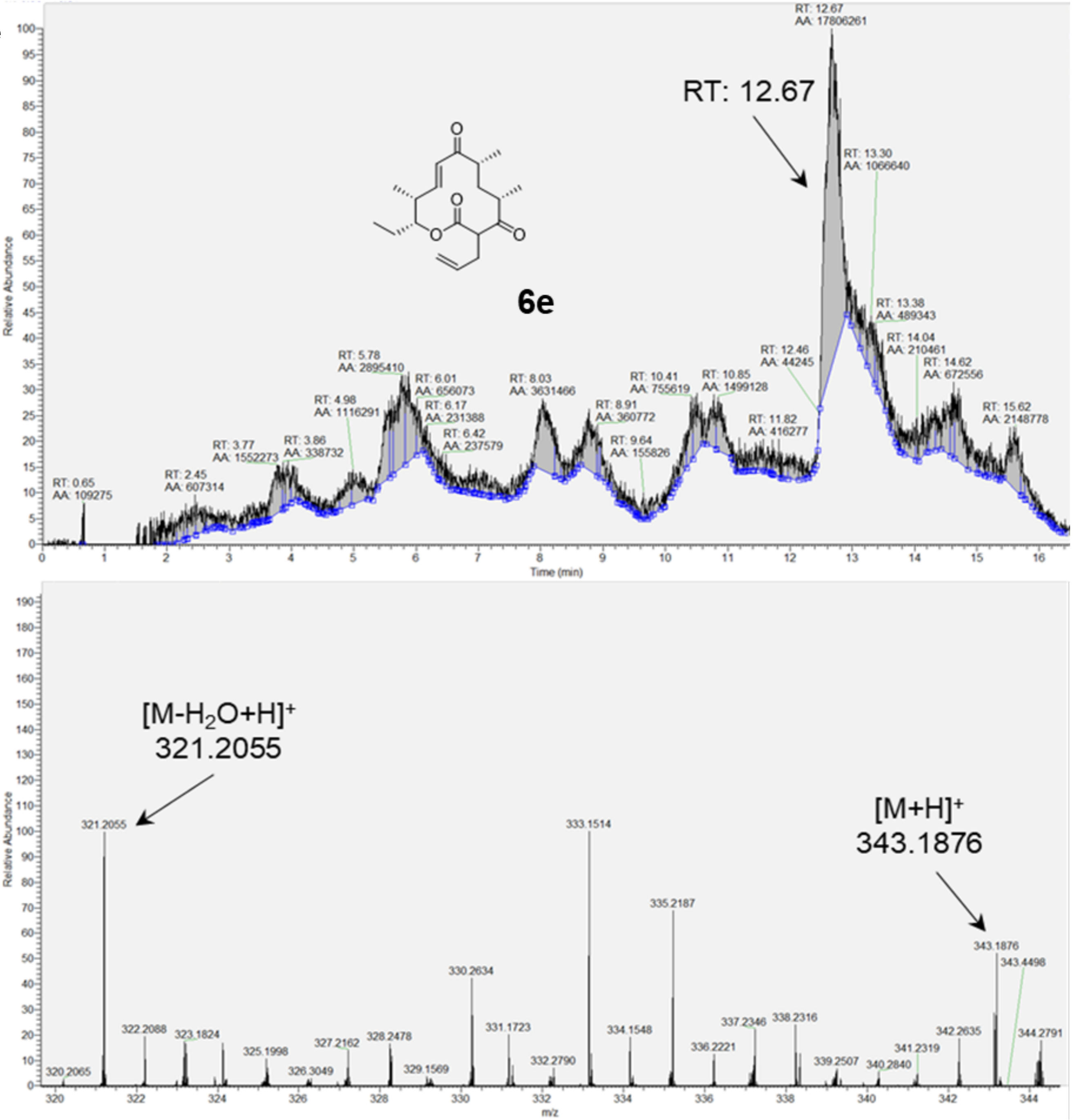
RT: 12.67
AA: 17806261
RT: 12.67
6e
RT: 13.30
AA: 1066640
RT: 13.38
AA: 489343
RT: 14.04
AA: 210461
RT: 14.62
AA: 672556
RT: 15.62
AA: 2148778
RT: 12.46
AA: 44245
RT: 11.82
AA: 416277
RT: 10.85
AA: 1499128
RT: 10.41
AA: 755619
RT: 9.64
AA: 155826
RT: 8.91
AA: 360772
RT: 8.03
AA: 3631466
RT: 6.42
AA: 237579
RT: 6.17
AA: 231388
RT: 6.01
AA: 656073
RT: 5.78
AA: 2895410
RT: 4.98
AA: 1116291
RT: 3.86
AA: 338732
RT: 3.77
AA: 1552273
RT: 2.45
AA: 607314
RT: 0.65
AA: 109275
Relative Abundance
Time (min)
[M-H2O+H]+
321.2055
[M+H]+
343.1876
321.2055
333.1514
335.2187
330.2634
343.1876
343.4498
322.2088
323.1824
325.1998
326.3049
327.2162
328.2478
329.1569
331.1723
332.2790
334.1548
336.2221
337.2346
338.2316
339.2507
340.2840
341.2319
342.2635
344.2791
320.2065
Relative Abundance
m/z

f

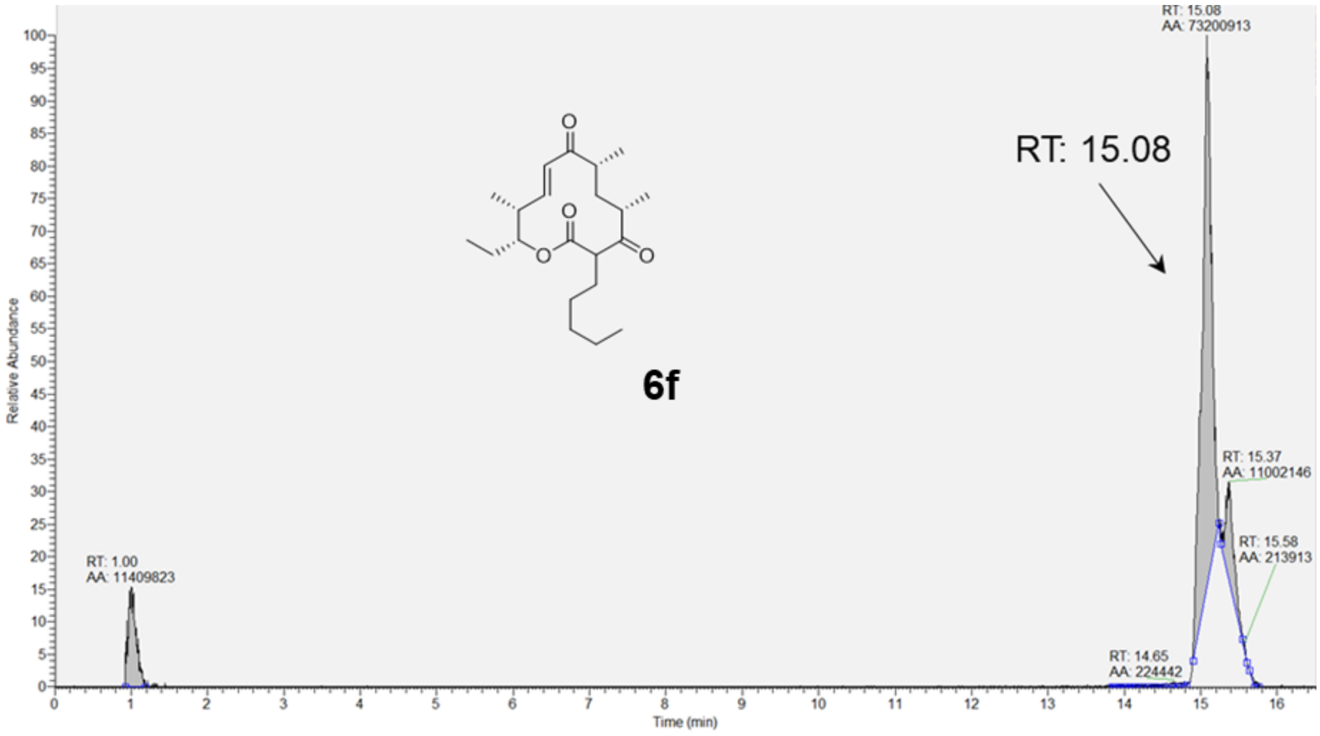

RT: 15.08
AA: 73200913
RT: 15.08
6f
RT: 15.37
AA: 11002146
RT: 15.58
AA: 213913
RT: 1.00
AA: 11409823
RT: 14.65
AA: 224442
Relative Abundance
Time (min)

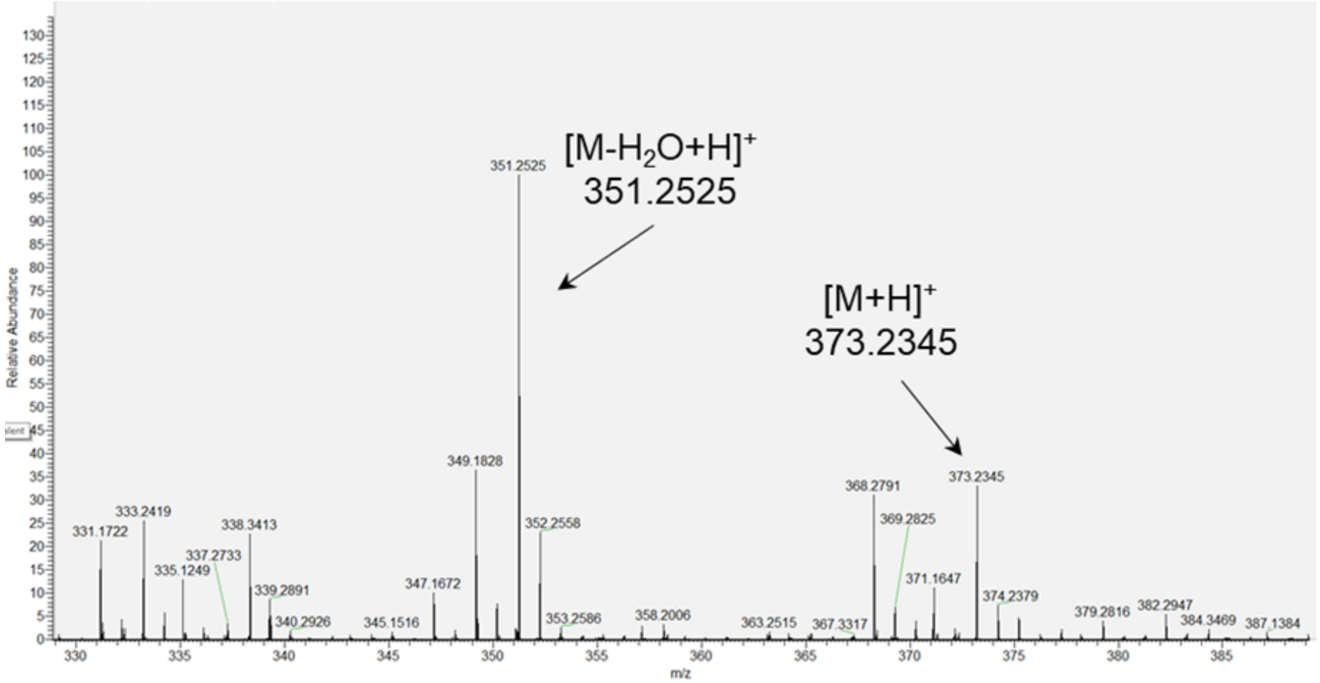

$[M-H_2O+H]^+$
351.2525
$[M+H]^+$
373.2345
351.2525
349.1828
368.2791
373.2345
333.2419
331.1722
338.3413
352.2558
369.2825
337.2733
335.1249
371.1647
339.2891
347.1672
374.2379
340.2926
345.1516
353.2586
358.2006
363.2515
367.3317
379.2816
382.2947
384.3469
387.1384
Relative Abundance
m/z

Supplementary Methods

Site-directed mutagenesis of Ery6TE

All Ery6 and Er6TE site-directed mutants were constructed by 'round-the-horn' mutagenesis using the templates from **Supplemental Table 7** and the oligonucleotide sequences described **Supplemental Table 8**.[1] Each PCR contained: 5X GC Phusion DNA polymerase buffer (4 µL), DNAase free water (11.8 µL), forward/reverse primer mix (2 µL, 10 µM), template DNA (1 µL, 50 ng/µL), dNTPs (0.4 µL, 2.5 mM each dNTP), DMSO (0.6 µL), and Phusion High Fidelity DNA Polymerase (0.2 µL) (New England Biolabs, NEB). PCR cycling parameters: step 1) 98 °C, 60 s; step 2) 29x [a) 98 °C, 10 s; b) 72 °C, 5 min]; step 3) 72 °C, 5 min. Next, the PCR mixture was subjected to restriction enzyme digest with *Dpn*I to remove any remaining template DNA. The *Dpn*I reaction mixture contained: 10X CutSmart Buffer (2 µL), the PCR mixture (17 µL), and *Dpn*I (1 µL). The mixture was vortexed, centrifuged, and incubated for 3 h at 37 °C. The digested reaction mixture was then purified by agarose gel electrophoresis, with a total of 8 µl DNA eluted at the final step. The PCR product was then ligated using T4 DNA ligase in a reaction containing: 1 µl of the 10X T4 DNA ligase buffer, 8 µl of the *Dpn*I-treated and purified PCR product, and 1 µl of the T4 DNA ligase (overnight incubation at 16 °C). Each subsequent ligation reaction was transformed directly into *E. cloni* 10G electrocompetent cells (Lucigen). Individual transformants were sequenced to identify incorporation of mutant codons.

Construction of Ery6TE motif chimeras

Motif swaps of Ery6TE AT were constructed using Gibson assembly (NEB) and boundaries defined by the Keasling group.[2] The Gibson assembly mixtures were used to transform *E. coli* DH5α competent cells. Successful incorporation of mutations was confirmed by DNA sequencing of purified plasmids from single transformants. Each mutant plasmid was transformed into *E. coli* K207-3 competent cells and plated onto LB agar plates (50 µg mL^{-1} kanamycin and 100 µg mL^{-1} spectinomycin).

Expression and purification of wild-type and mutant MatB

The expression and purification of MatB wild-type and T207G/M306I have been previously described.[3-5] Briefly, *E. coli* BL21(DE3) pLysS competent cells were transformed with plasmid. and positive transformants were selected on LB agar supplemented with 50 µg/mL kanamycin. A single colony was transferred to LB (3 mL) supplemented with kanamycin (50 µg/mL) and grown at 37 °C and 250 rpm overnight. The culture was used to inoculate LB media (1 L) supplemented with kanamycin (50 µg/mL). One liter culture was incubated at 37 °C and 250 rpm to an OD_{600} of 0.6, at which time protein synthesis was induced by the addition of IPTG to a final concentration of 1 mM. After incubation at 18 °C and 200 rpm for 18 h, cells were collected by centrifugation at 5,000 *g* for 20 min and resuspended in 100 mM Tris-HCl pH 8.0 (20 mL) containing NaCl (300 mM) and then lysed by sonication. Following centrifugation at 10,000 *g*, the soluble extract was loaded onto a 1 mL HisTrap HP column (GE Healthcare, Piscataway, NJ) and purified by fast protein liquid chromatography using the

following buffers: wash buffer [20 mM phosphate (pH 7.4) containing 0.5 M NaCl and 20 mM imidazole] and elution buffer [20 mM phosphate (pH 7.4) containing 0.5 M NaCl and 200 mM imidazole]. The purified protein was concentrated using an Amicon Ultra 30 kDa MWCO centrifugal filter (Millipore Corp., Billerica, MA) and stored as 10% glycerol stocks at -80 °C. Protein purity was verified by SDS-PAGE. Protein quantification was carried out using the Bradford Protein Assay Kit from Bio-Rad.

Synthesis of acyl-CoAs by MatB

The MatB-catalyzed synthesis of extender units **1** (wild-type MatB) and **2a-f** (MatB T207G/M306I) has been previously described.[3-5] Briefly, reactions were performed in a 50 µL reaction mixture containing 100 mM sodium phosphate (pH 7), $MgCl_2$ (2 mM), ATP (12 mM), coenzyme A (8 mM), malonate or corresponding analog (16 mM) and wild-type or mutant MatB (10 µg) at 25 °C. Aliquots were removed after 3 h incubation, and quenched with an equal volume of ice-cold methanol, centrifuged at 10,000 *g* for 10 min, and cleared supernatants used for HPLC analysis on a Varian ProStar HPLC system. A series of linear gradients was developed from 0.1% TFA (A) in water to methanol (HPLC grade, B) using the following protocol: 0-32 min, 80% B; 32-35 min, 100% A. The flow rate was 1 mL/min, and the absorbance was monitored at 254 nm using Pursuit XRs C18 column (250 x 4.6 mm, Varian Inc.). To ensure complete conversion, the malonate analogue and the acyl-CoA product HPLC peak areas were integrated, and the conversion (%) calculated as a percent of the total peak area. Product elution times and LC-MS data were in complete agreement with that previous described.[3,4]

Supplementary References

1 Moore, S. *'Round-the-horn site-directed mutagenesis*, <https://openwetware.org/wiki/'Round-the-horn_site-directed_mutagenesis>

2 Yuzawa, S. *et al.* Comprehensive in vitro analysis of acyltransferase domain exchanges in modular polyketide synthases and its application for short-chain ketone production. *ACS Synth. Biol.* **6**, 139-147 (2017).

3 Koryakina, I. *et al.* Poly specific trans-acyltransferase machinery revealed via engineered acyl-CoA synthetases. *ACS Chem. Biol.* **8**, 200-208 (2013).

4 Koryakina, I., McArthur, J. B., Draelos, M. M. & Williams, G. J. Promiscuity of a modular polyketide synthase towards natural and non-natural extender units. *Org. Biomol. Chem.* **11**, 4449-4458 (2013).

5 Koryakina, I. & Williams, G. J. Mutant malonyl-CoA synthetases with altered specificity for polyketide synthase extender unit generation. *ChemBioChem* **12**, 2289-2293 (2011).

6 Crooks, G. E., Hon, G., Chandonia, J. M. & Brenner, S. E. WebLogo: A sequence logo generator. *Genome Res.* **14**, 1188-1190 (2004).
